# High-performance photocatalytic reduction of Cr(VI) using a retrievable Fe-doped WO_3_/SiO_2_ heterostructure

**DOI:** 10.1186/s11671-023-03919-0

**Published:** 2024-01-31

**Authors:** Natkritta Boonprakob, Duangdao Channei, Chen Zhao

**Affiliations:** 1 Program of Chemistry, Faculty of Science and Technology, Uttaradit Rajabhat University, Uttaradit, 53000 Thailand; 2 Department of Chemistry, Faculty of Science, Naresuan University, Phitsanulok, 65000 Thailand; 3 School of Materials and Energy, Guangdong University of Technology, Guangzhou, 510006 People’s Republic of China

**Keywords:** Fe-doped WO_3_/SiO_2_, Heterostructure, Photocatalytic reduction, Nanocomposite, Cr^6+^

## Abstract

The enhancement of the photocatalytic performance of pristine WO_3_ was systematically adjusted due to its fast recombination rate and low reduction potential. A designed heterostructure photocatalyst was necessarily synthesised by Fe^3+^ metal ions doping into WO_3_ structure with and composition modification. In this study, we synthesised a retrievable Fe-doped WO_3_/SiO_2_ heterostructure using a surfactant-assisted hydrothermal method. This heterostructure was then employed as an effective photocatalyst for the removal of Cr(VI) under visible light irradiation. Enlarged photocatalytic reduction was observed over a synergetic 7.5 mol% Fe-doped WO_3_/SiO_2_-20 nanocomposite, resulting in dramatically increased activity compared with undoped WO_3_ and SiO_2_ nanomaterials under visible light illumination within 90 min. The presence of 7.5 mol% Fe^3+^ ion dopant in WO_3_ optimised electron–hole recombination, consequently reducing WO_3_ photocorrosion. After adding SiO_2_ nanoparticles, the binary WO_3_-SiO_2_ nanocomposite played roles as both adsorbent and photocatalyst to increase specific surface area. Thus, the 7.5 mol% Fe-doped WO_3_/SiO_2_-20 nanocomposite catalyst had more active sites on the surface of catalyst, and enhanced photocatalytic reduction was significantly achieved. The results showed 91.1% photocatalytic reduction over the optimum photocatalyst, with a photoreduction kinetic rate of 21.1 × 10^–3^ min^−1^, which was approximately four times faster than pristine WO_3_. Therefore, the superior optimal photocatalyst demonstrated reusability, with activities decreasing by only 9.8% after five cycles. The high photocatalytic performance and excellent stability of our photocatalyst indicate great potential for water pollution treatments.

**Supplementary Information:**

The online version contains supplementary material available at 10.1186/s11671-023-03919-0.

## Introduction

Stainless steel manufacturing has seen a rapid increase in the use of chromium (Cr). Most stainless steels contain approximately 18% Cr to harden and toughen the alloys and increase their corrosion resistance, especially when used at high temperatures [1]. This has, however, led to Cr contamination, affecting living organisms in both soil and aqueous environments [2]. Several methods have been employed to remove Cr^6+^ from industrial wastewater, including chemical and electrochemical precipitation, filtration, and ion exchange [3], prior to waste discharge. Among these, advanced oxidation processes (AOPs) are used in conjunction with semiconductor photocatalysts such as TiO_2_, ZnO, and WO_3_. These photocatalysts have been widely considered viable in the treatment of less hazardous organic pollutants, such as azo dyes, and heavy metal contaminants in wastewater effluents. Notably, WO_3_, an n-type semiconductor with a band gap energy of 2.9–3.2 eV [4], is widely used in photocatalysis owing to its low costs, eco-friendliness, excellent physicochemical stability, electrochromic properties, and ease of synthesis [5–7]. Therefore, its use in visible-light-harvesting photocatalysis has become a major focus of research [8, 9]. Various types of controlled WO_3_ morphologies incorporating single transition metal dopants or two metals as co-dopants can be obtained via different synthetic techniques—these semiconductors (offering different band gap energies) can be combined with supporting materials, such as mesoporous SiO_2_ nanoparticles (NPs), to form heterostructures that raised the light-harvesting ability of the photocatalyst [10]. In the past decade, several researchers have reported on the superior performance of metal-ion-doped WO_3_ photocatalysts and (material-supported) W-/metal oxide-based heterostructures in the photocatalytic degradation of wastewater pollutants, including various organic dyes [11–14] and toxic Cr^6+^ contaminants, [15–18], hydrogen production [19, 20], and enhanced gas-sensing performance [21]. Considering the above-mentioned literature sources and the advantages and drawbacks of photocatalysts based on WO_3_, metal doped WO_3_, and heterojunctions with SiO_2_, this work aimed to improve upon the WO_3_ photocatalyst, implementing heterojunction modifications of the WO_3_ semiconductor by blending with SiO_2_ NPs and further doping with Fe^3+^ ions to improve the photocatalytic reduction efficiency of Cr^6+^. Notably, SiO_2_ NPs display high purity and high specific surface areas of 175–225 m^2^ g^−1^, not only facilitating the adsorption of organic compounds but also assisting in the dispersal of metal oxide particles [22]. Therefore, the SiO_2_-supported W-based or WO_3_ heterostructure photocatalyst in our above-mentioned research has shown superior performance over other photocatalysts in both energy and environmental applications [23], such as the photocatalytic degradation of herbicides and organic dyes and the removal of Cr^6+^ via photoreductive conversion into the less toxic Cr^3+^ [24]. Recently, nanosized SiO_2_ has been utilised in nanomaterial fabrication and semiconductor modification. Further, SiO_2_ NPs have been used as a support material for W-based photocatalysts due to their cost-effectiveness, easy availability, high mechanical stability, and transparency in the excitation spectral range of WO_3_ [X]. To the best of our knowledge, our present work is novel in terms of the iron dopant percentage/loading and the combination of a SiO_2_ support with high-purity WO_3_, yielding a reproducible method of photocatalyst synthesis for the photoreduction of the hazardous Cr^6+^ to the less toxic Cr^3+^ [25]. The superior reusability of the optimal catalyst after five cycles reveals its high efficiency and stability. Moreover, the Fe-doped WO_3_/SiO_2_ heterostructure photocatalyst was synthesised via the notably inexpensive CTAB-assisted hydrothermal technique, exhibiting enhanced photocatalytic potential for wastewater treatment under visible light irradiation from a very low-intensity and low-cost halogen lamp.

Thus, the heterostructure Fe-doped WO_3_/SiO_2_ nanocomposites were synthesised via hydrothermal treatment with Fe^3+^ doping in this study. The weight ratio of Fe-doped WO_3_ to SiO_2_ was varied. The physicochemical properties of the prepared samples were characterised to describe the effects of the promoted photocatalytic removal of Cr^6+^. Additionally, the photocatalytic reduction of a toxic Cr^6+^ solution over the prepared retrievable nanocatalyst was evaluated under visible light illumination (*λ* > 400 nm), which determined the kinetic rate of Cr^6+^photoreduction. In addition, the photocatalytic reduction efficiencies (%PE) and kinetic rate (*k*_*t*_) values were also evaluated and compared under the effect of catalyst dosage and the pH of the suspension. Furthermore, the cycling ability of the optimum photocatalyst was also investigated.

## Experiments

### Chemicals

All chemicals used in our studied are the analytical reagents (AR) grade and used without further purification. Sodium tungsten dihydrate (Na_2_WO_4_·2H_2_O, 99.0%, Sigma-Aldrich), and Iron (III) chloride (FeCl_3_, 99.0%, Loba Chemie), cetyltrimethylammonium bromide (CTAB, 98%, Sigma-Aldrich), Rhodamine B (C_28_H_31_ClN_2_O_3_, 99.0%, Loba Chemie), and a chromium (VI) ICP-grade standard solution (99.9%, Sigma-Aldrich) were used. The remaining chemicals and solvents were of purified SiO_2_ (99.5%) powder (10–20 nm) purchased from Sigma-Aldrich., the Milli Q (Millipore), ultrapure water, or deionised (DI) water having a resistivity of 18.2 MΩ.cm at room temperature and conductivity of 0.054 μS were utilised through the experiments.

### *Synthesis of Fe-doped WO*_*3*_*/SiO*_*2*_* heterostructure NPs*

**Fig. 1:**
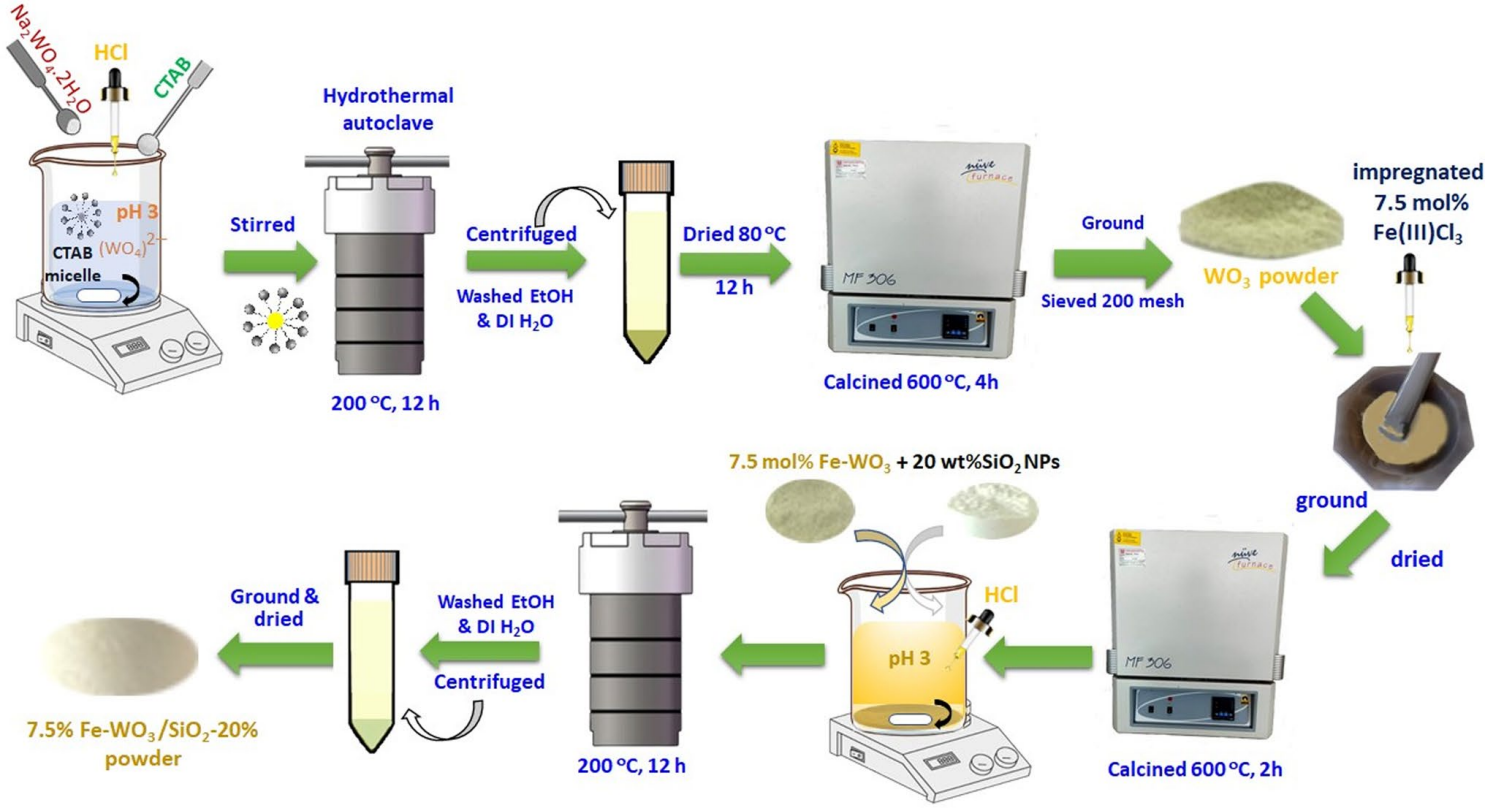
Schematic illustration of the synthesis procedure of 7.5%Fe-WO_3_-SiO_2_-20 nanocatalyst

Pure WO_3_, Fe-doped WO_3_, and Fe-doped WO_3_/SiO_2_ heterostructures were prepared via a surfactant-assisted hydrothermal and impregnation procedure. First, monoclinic WO_3_ nanostructures were synthesised using a CTAB-assisted hydrothermal method. Briefly, a certain amount of Na_2_WO_4_⋅2H_2_O was dissolved in DI water with constant stirring to obtain a clear solution (A). Subsequently, a 0.17 M aqueous CTAB solution was prepared and slowly added to solution (A). The conc. HCl solution was then applied dropwise for pH adjusted to 3.0 ± 0.3 using under vigorously stirring. Thereafter, the mixture was transferred into a Teflon-lined stainless-steel autoclave and kept at 200 °C for 12 h. The precipitate was washed several times and dried at 80 °C for 12 h using an air oven. Finally, the synthesised powder was calcined at 600 °C. The Fe-doped WO_3_ NPs were prepared via wet impregnation using Fe(III)Cl_3_ metal ions precursor, with doping concentrations of 2.5, 5.0, 7.5, and 10.0 mol% of Fe^3+^ contents. The solutions with different concentrations of Fe^3+^ solution were each added to continuously ground, dried, and sintered at high temperature [26]. Initially, the optimum Fe-doped WO_3_ sample was evaluated via the photoreduction of Cr^6+^ prior to prepare the Fe-WO_3_/SiO_2_ nanocomposite. The 7.5 mol% Fe-doped WO_3_ sample displayed the highest photoreduction efficiency as shown in the supporting information (Fig. S1). Therefore, it was used in the further fabrication of the Fe-WO_3_/SiO_2_ heterostructure catalyst. Figure 1 schematically demonstrates the synthesis process of the 7.5% Fe-WO_3_/SiO_2_-20 composite catalyst nanoparticles. The nominal weight ratio of 7.5 mol% Fe-doped WO_3_:SiO_2_ was set at 50:50 and 80:20 wt%. The samples were prepared by directly mixing stoichiometric amounts of Fe-doped WO_3_ and commercial SiO_2_ nanopowder (99%, Sigma-Aldrich). The stoichiometric SiO_2_ powder was sonicated in 90 mL of DI water and then directly mixed with 7.5 mol% Fe-doped WO_3_ with stirring to obtain a homogeneous mixture with pH adjust of 3.0 by using HCl solution. The mixture was poured into a 350-mL Teflon-line autoclave and kept at 200 °C for 12 h. The precipitates were separated, milled to acquire a dried powder. The calcination characteristics of both ratios of the Fe-doped WO_3_/SiO_2_ nanocomposites were investigated at same ambient mention above. The obtained photocatalysts were denoted to SiO_2_, WO_3_, 7.5% Fe-WO_3_, 7.5% Fe-WO_3_/SiO_2_-50, and 7.5% Fe-WO_3_/SiO_2_-20.

### Characterisation

X-ray diffractometer (XRD, PANalytical X’Pert PRO MPD, UK) with *λ* of Cu *K*_*α*_ radiation (1.541 Angstrom) at 40 kV/30 mA was used for determining the phase confirmation and crystal structures of the as-synthesised NPs samples. The detector was detected a 2*θ* range of 10–70^ο^ with 0.04° s^−1^ of scanning rate. Field emission scanning electron microscope (FE-SEM; JEOL JSM 7800F, Japan) was investigated to capture 3D shapes, and particles micrograph, and size of sample. In addition, energy-dispersive X-ray spectroscope (EDX, Horiba–Hitachi, Japan) integrated with of SEM was indicated the dispersing of elements composition. The high-resolution transmission electron microscope (HRTEM; Tecnai G20, USA, 200 kV acceleration) was defined the microstructural details particularly including the precise sizes of 2D NPs, morphology, and polycrystalline nanostructure of the samples. A diffuse reflectance UV–visible spectrophotometer (DRS-UV–Vis spectrophotometer, Shimadzu 3101-PC, Japan) combined with integrating sphere (ISR-260) was recorded the DRS spectra against BaSO_4_ at *λ* of 200–1200 nm. X-ray photoelectron spectroscope (XPS; PHOIBOS analyser, Germany) was utilised to evaluate the surface oxidation states and chemical composition of the obtained materials by applying Al *K*_*α*_ radiation at 1400 eV. All absolute binding energy (B.E.) of XPS spectra was calibrated by C 1*s* at the peak of 284.6. A N_2_ gas adsorption equipment analyser (Micromeritics 3FleX, USA) was determined the Brunauer–Emmett–Teller (BET) surface area, pore volumes, and pore sizes at the vacuum condition after the degassing of the sorption test. The photoluminescence (PL) spectrophotometer excited with 345 and 525 nm light sources were used for analysing the emission wavelength (PerkinElmer LS-45 photoluminescence, USA) manipulated to report PL spectra of the NPs semiconductors. A zetasizer (ZS Malvern, UK) was used to measure the point of zero charge (PZC) of the optimal photocatalyst.

### Photocatalytic reduction experiments

Following the standard procedure, 0.10 g of the catalyst was suspended in 100.0 mL of 20.0 mg·L^−1^ Cr^6+^ solution (ICP grade). The solution’s pH was then adjusted to 3.0 using 0.10 M HClO_4_ and 0.10 M NaOH. Prior to light exposure, the mixture was continuously stirred in the dark for 30 min to reach adsorption–desorption equilibrium. Subsequently, the solutions were irradiated using a 50-W halogen lamp (12 V, Phillips, Netherlands) with a light intensity of 364 W.m^−2^. At specific time intervals, 4.0 mL of the reaction suspension was withdrawn and filtered through a 0.22-μm micro-syringe nylon membrane (Pro Filter, China). The concentration of Cr^6+^ after reduction was determined colourimetrically using 1,5-diphenylcarbazide (99.0%, Loba Chemie) by measuring the characteristic UV–Vis absorption peak at 554 nm, detected by a PG-92 + UV–Vis spectrophotometer (Germany). The %PE was calculated using Eq. 1 in our previous work [27]:

1 $$\% \, PE = \frac{{C_{0} - C_{t} }}{{C_{0} }} \times 100$$

where *C*_0_ is the initial concentration (mg L^−1^), and *C*_*t*_ is the concentration at time (mg L^−1^). The photocatalytic kinetic reaction was investigated using the pseudo-first-order kinetic model of Eq. 2 as follows:

2 $$\ln \frac{{C_{0} }}{{C_{t} }} = k_{t}$$

where *k*_*t*_ represents the apparent pseudo-first-order rate constant (min^−1^).

Furthermore, the reusability of superior nanocomposite catalyst compared with other NPs catalysts was also evaluated towards the photoreduction of Cr^6+^ solution for 5 cycles under the same ambient. In each cycle, to investigate the stability of the binary NPs catalyst. The non-magnetic sample was centrifuged the supernatant after first cycle run, sedimented, and washed by. After each cycle, the catalyst particles were recovered by centrifugation and then washed with 95% ethanol and hot water DI water (70 °C) for 3 several times [28]. The photocatalyst was then dried at 60 °C for 12 h in an air oven and then ground to a fined power. Subsequently, the catalyst was immersed and dispersed into a fresh Cr^6+^ solution prior to the next run. After 5 cycle tests, the dried precipitate photocatalyst had demonstrated the stability and recyclability against the chemical and photocorrosive property. The structural and phase crystallinity of reused catalyst was confirmed by the FE-SEM and XRD characterisation. In addition, Inductively Coupled Plasma-optical emission spectroscope (ICP-OES; PerkinElmer-4300 DV) was confirmed to determine Fe ions leaching in the supernatant Cr^6+^ solution.

## Results and discussion

### Crystal structure

**Fig. 2:**
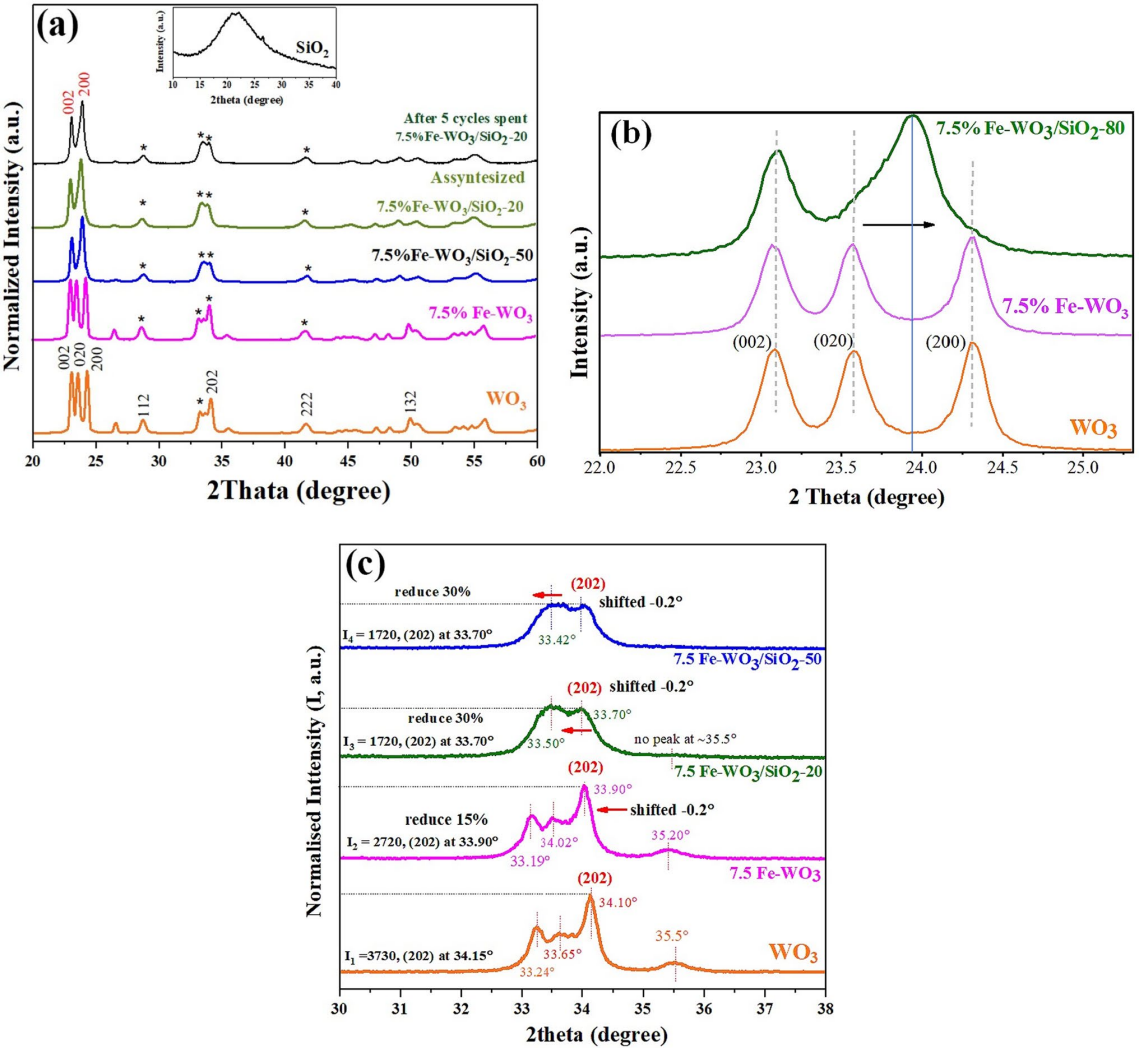
**a** X-ray diffraction (XRD) patterns of overall nanocatalyst samples, **b** magnification of the XRD patterns in the 2*θ* range of 22.0°–25.0°, and **c** XRD patterns in the 2*θ* range of 30.0°–38.0°

The XRD patterns of all synthesised photocatalysts are shown in Fig. 2a, with the broad peak at 22.19° corresponding to amorphous SiO_2_ (JCPDS No. 39–1425) [29]. The XRD pattern of WO_3_ exhibited characteristic peaks at 23.08°, 23.58°, 24.31°, 28.08°, 34.12°, and 49.80°, which could be assigned to the (002), (020), (200), (122), (202), and (132) crystalline planes of monoclinic WO_3_ (JCPDS No. 43–1035) [30]. Moreover, when loading nanosized SiO_2_ into the WO_3_ crystal structure, the three main peaks of (002), (020), and (200)–of WO_3_ were deconstructed and broadened. Consequently, there were only two peaks at 23.10° while the Fe-WO_3_ doping sample shifted to the lower angle (− 0.2°). The radii compared with the pristine WO_3_ pattern could be noticed, as illustrated for 7.5% Fe-WO_3_/SiO_2_-20 (Fig. 2b). Additionally, Fig. 2c illustrates the XRD pattern of the (020) peak of WO_3_ (2*θ* ~ 33.2°–34.1°). The peaks split into three peaks, and the (202) peak was aligned at 34.10 while the Fe-WO_3_ doping sample shifted to the lower angle (− 0.2°). The deviation values of the Fe-WO_3_ samples decreased towards lower angles compared with the pristine WO_3_ pattern. This could be attributed to the ionic radii of the lattice coordinated Fe^3+^ and W^6+^ ions and the substitution of the latter with the former because the ionic radii of Fe^3+^ (64 pm) and W^6+^ (62 pm) were very similar [31]. In the meantime, the two broad peaks of the nanocomposite catalysts (20% and 50%) greatly collapsed, causing the (202) peak’s strength to decrease by 10%. Furthermore, when SiO_2_ was added, the XRD diffractogram of Fe-WO_3_/SiO_2_-20 showed noticeably less crystallinity than the pristine monoclinic WO_3_ diffraction pattern. As 20% and 50% SiO_2_ loading into the WO_3_ crystal structure occurred, it was evident that the normalised intensities of various peaks collapsed and disappeared, generating the emerging phase. The presence of SiO_2_ NPs combination was noticeable in the X-ray diffraction patterns of the Fe-WO_3_, Fe-WO_3_/SiO_2_-20, and Fe-WO_3_/SiO_2_-20 composite samples, which demonstrated a decrease in crystallinity degree and a lower peak intensity in the overall diffraction peaks [32]. Consequently, XRD diffraction peak might be attributed to the emergence of recrystallisation between amorphous SiO_2_ NPs and WO_3_. Moreover, it supposes that the peaks disappeared, the intensities decreased, and that broadening peaks at 2*θ* of approximately 23.9° and 33.42°, respectively, formed and initiated the combination of two broadening peaks (Fig. 2b and c). This possibility of SiO_2_ covering intensity reduced, and some peaks disappear and might be formed to the new phase. Secondly, there is the possibility for SiO_2_ to impregnate the WO_3_ surface because the amorphous SiO_2_ particles might interact or disturb the lattice planes of WO_3_ crystal structure [33]. The crystallite sizes of the obtained samples calculated from Scherer’s equation [34] are shown in Table 1. Specifically, Fe-WO_3_/SiO_2_-50, Fe-WO_3_/SiO_2_-20, Fe-WO_3_, WO_3_, and SiO_2_ products have crystallite sizes of 16.4, 18.8, 21.7, 24.3, and 6.47 nm, respectively. It is evident that Fe-WO_3_/SiO_2_-20 has a drastically smaller scale compared to pure WO_3_. This observation supported the above assumption that the loading of Fe^3+^ ions and SiO_2_ can inhibit WO_3_ grain growth, thereby resulting in a smaller crystalline particle size [35].

**Table 1:** Summary of average crystallite size, band gap (*E*_*g*_), specific surface area (SSA_**BET**_), pore volume, and atomic weight of Fe content (at%) from EDS spectra of prepared photocatalysts

Photocatalyst	Crystallite size (nm)	*E*_*g*_ (eV)	SSA_BET_ (m^2^ g^−1^)	Pore volume (cm^3^ g^−1^)	Fe content* (at%)
SiO_2_	6.47	4.19	138.1	1.055	–
WO_3_	24.3	2.83	7.68	0.043	–
7.5% Fe-WO_3_	21.7	2.76	8.39	0.064	3.20
7.5% Fe-WO_3_-20	18.8	2.70	50.4	0.227	4.16

*Value from EDS spectra of obtained nanocatalyst samples from SEM–EDS

### DRS and UV–Vis spectra study

**Fig. 3:**
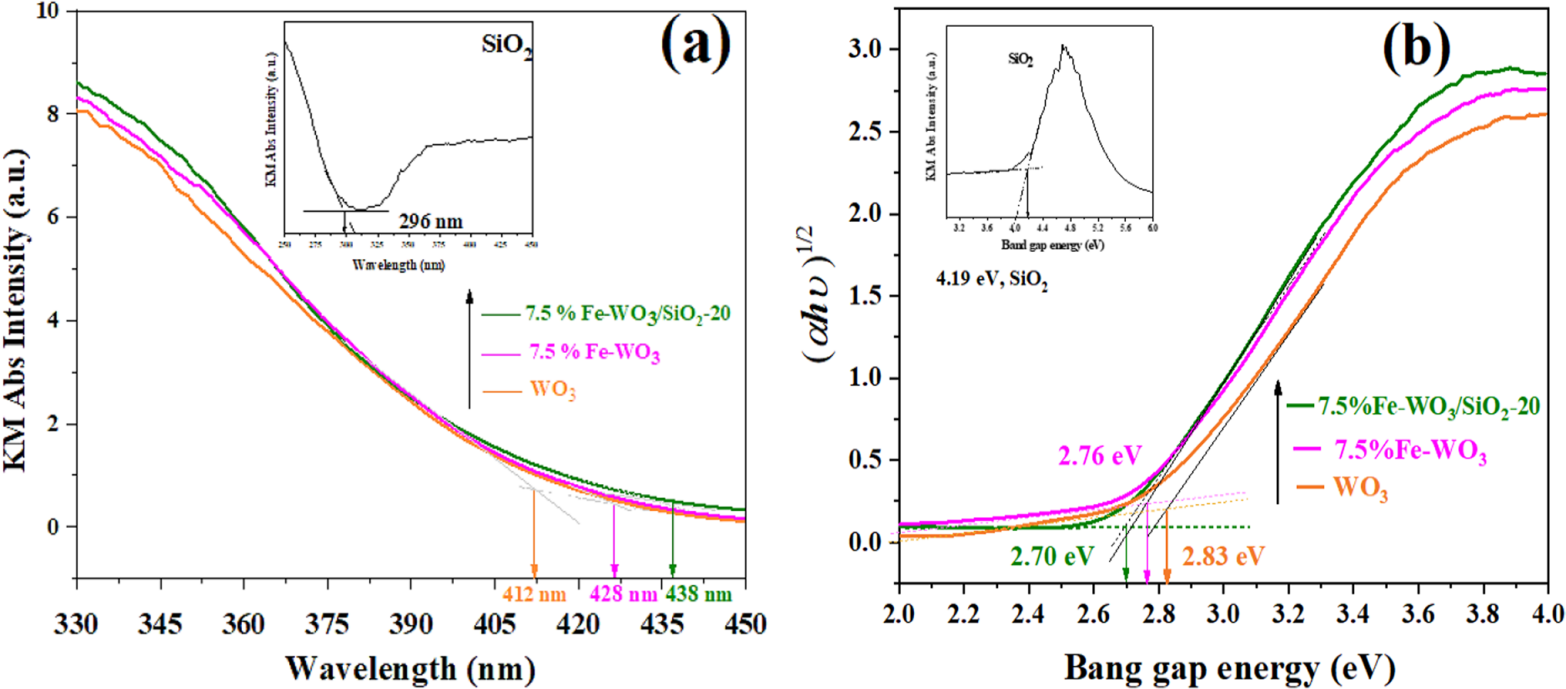
**a** KM absorption spectra **b** band gap energies of WO_3_, Fe-WO_3_, and Fe-WO_3_/SiO_2_-20 and SiO_2_ (inset) photocatalysts, respectively

The Kubelka–Munk (KM) absorption spectra of bare WO_3_, Fe-WO_3_, and Fe-WO_3_/SiO_2_-20 photocatalysts are shown in Fig. 3. A small shift at the absorption onset and broadening of the absorption tail towards visible light region were observed upon doping WO_3_/SiO_2_ with Fe^3+^. The KM absorption spectra were calculated using Eq. 3:

3 $$F(R_{\infty } ) = \frac{{(1 - R_{\infty } )^{2} }}{2R}$$

where *R*_*∞*_ is the absolute diffuse reflectance for an infinitely thick sample. The UV–Vis DRS spectrum of amorphous SiO_2_ exhibited very weak absorption intensity (Fig. 3a inset), whereas the ionic WO_3_ NPs samples displayed high absorption intensity, as shown in Fig. 3a. The band gap energy (*E*_*g*_) of the catalysts can be obtained as illustrated in Fig. 3b and calculated using Eq. 4 as our latest work as follows:

4 $$(\alpha h\upsilon )^{{{1}/{2}}} = {\text{ A }}(h\upsilon - \, E_{g} )$$

where *α* is linear absorption coefficient, *h* is the Planck’s constant, *υ* is light frequency, A is the proportionality constant, and *E*_*g*_ is the band gap energy. The power of parenthesis is ½ for direct band gap WO_3_ semiconductor, respectively, reported in Table 1.

Attribution to intrinsic to the band gap absorption can be determined by a linear extrapolation to the energy axis as shown in Fig. 3b. From the band gap energy values obtained by the intercept X–Y of the DR-UV–Vis spectra (Fig. 3b) were *ca.* 2.83, 2.76, 2.70 eV for WO_3_, Fe-WO_3_, and Fe-WO_3_/SiO_2_-20, respectively. The lowest band gap energy of photocatalyst is received from 7.5% Fe-WO_3_/SiO_2_-20 (2.70 eV). The significant redshift of the light absorption range upon added SiO_2_ and Fe^3+^ metal ions amount in the nanocomposite samples corresponds well with the reduced band gap energy. The7.5% Fe-WO_3_ and 7.5% Fe-WO_3_/SiO_2_-20 band gap decrease of *ca.* 0.7 and 1.3 eV compared with the pure WO_3_, respectively. These results can be described the effect of Fe^3+^ ion doping into the WO_3_ crystal structure on the photocatalytic samples, indicating the visible light response of these samples, which led to the abundant generation of electron–hole pairs and, consequently, improved photocatalytic activity [36]. Moreover, SiO_2_ might be disturbed Fe-WO_3_ crystal structure resulting in the drastically decreased the *E*_*g*_ of nanocomposite sample. The calculated crystallite sizes, band gaps, and textural properties of the prepared samples.

**Fig. 4:**
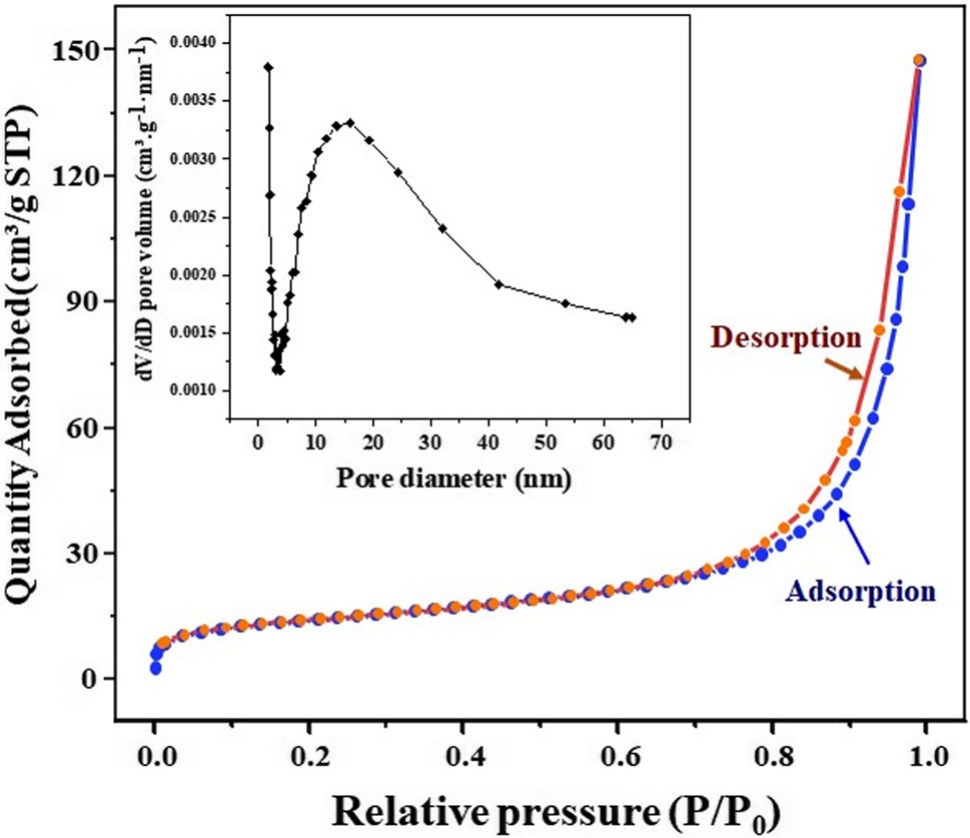
N_2_ absorption–desorption curves of 7.5% Fe-WO_3_-SiO_2_-20 nanocomposite inset of pose size distribution

In addition, the N_2_ absorption–desorption curves of the 7.5% Fe-WO_3_/SiO_2_-20 nanocomposite and the corresponding pore size distribution are shown in Fig. 4. The isotherms of the Fe-doped WO_3_ nanostructures can be categorised as a typical type IV [37], with hysteresis loops observed in the relative pressure *(P/P*_*0*_*)* of 0.99. The total pore volume of the 7.5% Fe-WO_3_/SiO_2_-20 sample was calculated as 0.227 cm^3^ g^−1^. The pore size distribution of 7.5% Fe-WO_3_/SiO_2_-20 obtained from the adsorption branch was placed at ~ 16.0 nm, as shown in the inset of Fig. 4. The 7.5% Fe-WO_3_/SiO_2_-20 catalysts were confirmed to be mesoporous materials. SiO_2_ exhibited a very high BET SSA_BET_ of 138.10 m^2^ g^−1^, whereas the undoped WO_3_ had the lowest value of 7.68 m^2^·g^−1^ among all the prepared NP products. However, the SSA_BET_ of 7.5% Fe-WO_3_/SiO_2_-50 became smaller than that of 7.5% Fe-WO_3_/SiO_2_-20. As shown in Table 1, the crystallite size of pure WO_3_ was larger than either 7.5% Fe-WO_3_, Fe-WO_3_/SiO_2_-20, or Fe-WO_3_/SiO_2_-50. After doping with Fe^3+^, the SSA_BET_ of 7.5% Fe-WO_3_ improved slightly to 8.39 m^2^ g^−1^. Incorporating 20 wt% of SiO_2_ into Fe-WO_3_ significantly increased the SSA_BET_ to 50.38 m^2^ g^−1^; however, further addition of SiO_2_ to 50 wt% decreased the SSA_BET_ to 21.29 m^2^ g^−1^. It might be possibly due to SiO_2_’s ability inhibited WO_3_ grain growth and disturbing the crystalline structure of monoclinic WO_3_ resulting in smaller crystalline particles upon loading Fe^3+^ and SiO_2_ [38]. The results suggested that the optimum loading of SiO_2_ was 20 wt%, as the large specific surface area, the larger pore volume than pure WO_3_, and the small particle size could enhance the adsorption of Cr^6+^. Thus, the raising Cr^6+^ photocatalytic removal performance had revealed [39].

### Morphology and microstructure

**Fig. 5:**
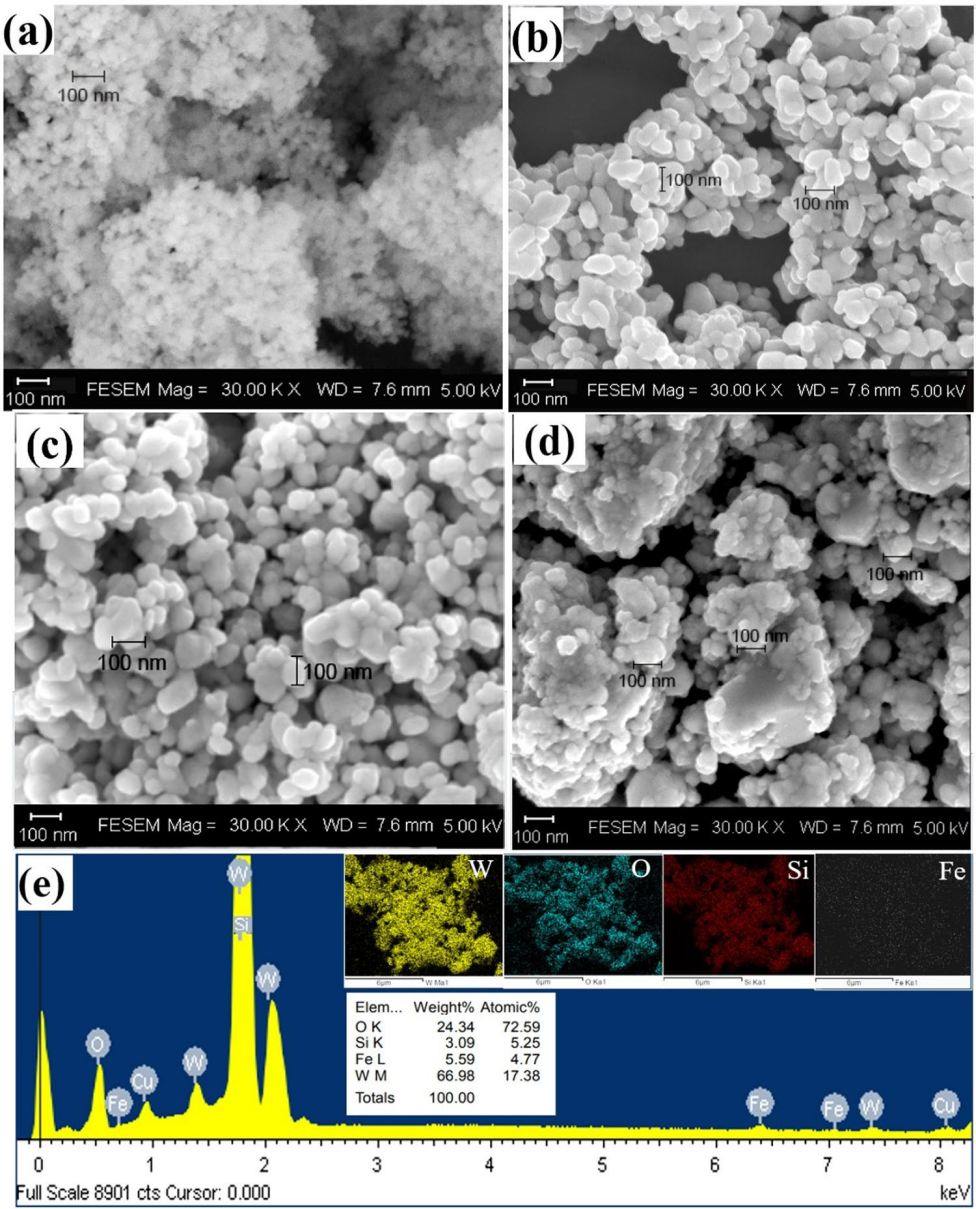
SEM micrographs of **a** SiO_2_, **b** WO_3_, **c** **7.5%** Fe-WO_3_, and **d** the **7.5%** Fe-WO_**3**_/SiO_**2**_**-20** nanocomposite; and **e** EDS spectrum and elemental mapping of (**d**)

**Fig. 6:**
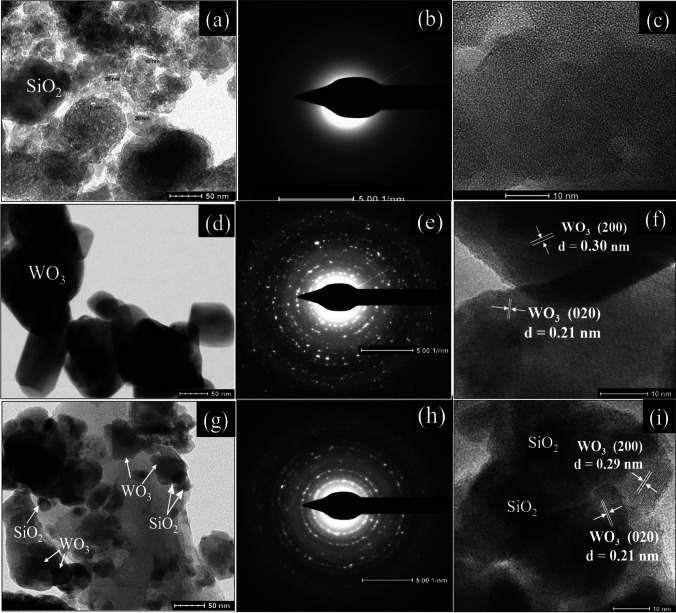
TEM, SAED, and HRTEM images of **a**–**c** SiO_2_, **d**–**f** WO_3_, and **g**–**i** 7.5% Fe-WO_3_/SiO_2_-20 NPs nanocomposite

The morphologies and microstructures of selected nanomaterials were imaged as (i) SEM and EDS as shown in Fig. 5a–e and (ii) TEM micrographs in Fig. 6a–i, respectively. The microstructures of the samples are shown in Fig. 5. An agglomeration of small SiO_2_ NPs is distinguished in the SEM image in Fig. 5a, whereas irregular rod-like WO_3_ NPs, with an average diameter of 50 nm, can be seen in Fig. 5b. Figure 5e displays the EDS spectra of the 7.5% Fe-WO_3_/SiO_2_-20 nanosized catalyst, with the elements W, Si, O, and Fe presenting as expected. Regarding elemental distribution, a binary 7.5% Fe-WO_3_/SiO_2_-20 heterojunction nanocomposite comprising the elements described above was homogeneously distributed in the sample. These results indicate the coexistence of Fe, as well as intimate contact between the component materials, confirming the presence of a binary Fe-WO_3_/SiO_2_ heterojunction. The SAED and HRTEM images in Fig. 6b–c reveal the non-crystalline nature of SiO_2_, agreeing with its broad XRD peak in Fig. 2a. By contrast, WO_3_ (Fig. 5d) exhibited a rod-like shape, with particle sizes in the range of 60–80 nm. Referring to Fig. 6d–f, the polycrystalline characteristic of WO_3_ was confirmed from the SAED ring pattern and lattice fringes of 0.21, 0.20, and 0.30 nm belonging to the (020), (200), and (110) crystal planes of monoclinic WO_3_, respectively. Pore size is a component of scaffolds, with the pores providing a region to which cells can adhere or become attached (EDS) [40]. Furthermore, TEM and HRTEM micrographs, as well as SAED patterns (Fig. 6), were obtained to reveal the particle sizes and internal microstructures of the samples (Fig. 6a–f). Regarding 7.5% Fe-WO_3_/SiO_2_-20 NPs (Fig. 6g–i) and SiO_2_ embedded in irregular WO_3_ NPs, electron diffraction patterns (SAED) revealed the particle sizes and internal microstructures of the NPs of WO_3_, with an average diameter of 10–20 nm.

### XPS analysis

**Fig. 7:**
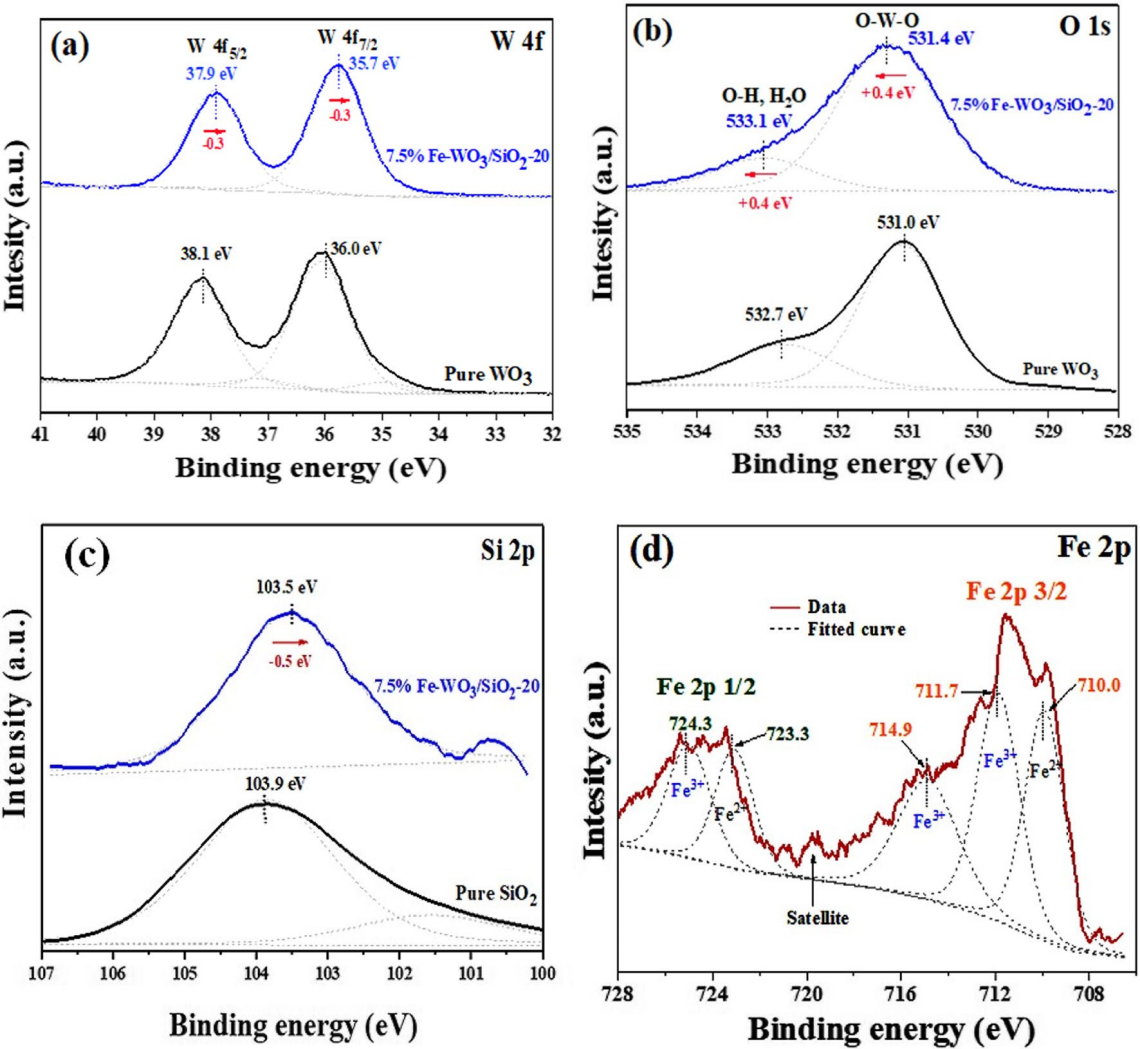
XPS spectra of **a** W 4f, **b** O 1*s*, **c** Si 2*p*, and **d** Fe 2*p* orbitals for pure WO_3_, SiO_2_, and 7.5% Fe-WO_3_/SiO_2_-20 nanocomposite

XPS analysis was investigated to obtain the oxidation states and chemical composition of WO_3_, pure SiO_2_, and 7.5% Fe-WO_3_/SiO_2_-20 nanostructures. Two intense spectra of the W 4f core level of pure WO_3_ and 7.5% Fe-WO_3_/SiO_2_-20 are exhibited in Fig. 7a. As for pure WO_3_, Fig. 7a displays both main peaks shown at 38.1 and 36.0 eV, corresponding to 4f_5/2_ and 4f_7/2_ [41], respectively. A shift of *ca.* 0.2 − 0.3 eV towards the lower B.E. was observed for the Fe-doped WO_3_/SiO_2_ sample. Therefore, this negative shift might have occurred from the increased in electron density due to the substitution of W^6+^ by Fe^3+^ ions [42]. The B.E. of O 1*s* of pristine WO_3_ and Fe-WO_3_/SiO_2_-20 nanocomposite (Fig. 7b) was assigned to two spectra in the comparison purpose. XPS spectra of W 4f and O 1*s* between the single-phase WO_3_ (W–O–W) and WO_3_/SiO_2_ nanocomposite indicated the different positions of B.E. peaks of couple samples, possibly due to the formation of the W–O–Si bond [43]. A similar negative shifted of the O 1*s* peak was also observed attributed to the rise of the lower-level occupancy of oxygen conduction band [44]. Figure 7c represents the single Si 2*p* peaks at 103.9 eV for pure SiO_2_, corresponding to Si^4+^ [45]. The lattice oxygen species at *ca.* 531–532 eV [46] and the adsorbed water/hydroxyl species at *ca.* 532–533 eV [47] exhibited a negative shift to ~ 530.5 eV in the Fe-WO_3_/SiO_2_ catalyst, as revealed in Fig. 7c [48]. Figure 7d reveals the quite spectrum for Fe 2*p* in Fe-WO_3_/SiO_2_. The predominant peaks of 2*p*_1/2_ occurred at B.E. of 724.3 eV whereas 2*p*_3/2_ marked at 711.7 eV, respectively [49]. The three peaks between 708.0 and 718.0 eV were deconvoluted to Fe 2*p*_3/2_. Lattice Fe^3+^ ions located at the peaks of ~ 710.0, 711.7, and ~ 714.9 eV, whereas for the Fe^2+^ ions peaks lied at 710.0 eV (2*p*_3/2_) and 724.3 eV (2*p*_**1/2**_) in Fe_2_O_3_ [50], respectively. The B.E. shift of XPS results could be ascribed to the divergent values between of Fe and W electronegativity (EN) [51]. For the EN of Fe and W comparison, compared with the Fe–O–Fe bond, bonding between Fe and O could have been slightly enhanced in the Fe–O–W bond, hence resulting in the increase the redshift B.E. of the Fe^3+^ peaks. Conversely, the bonding between W and O was slightly decreased in Fe–O–W opposed to W–O–W. From aforementioned, XPS peak shifts slightly because W^6+^ cations were replaced by Fe^3+^. Thus, Fe–O–W bond was formed. Particularly, the metal doping and intercalated doping in WO_3_ express enhanced crystal structure in preference to surface modification [52].

### Photocatalytic reduction activities and kinetics studies

**Fig. 8:**
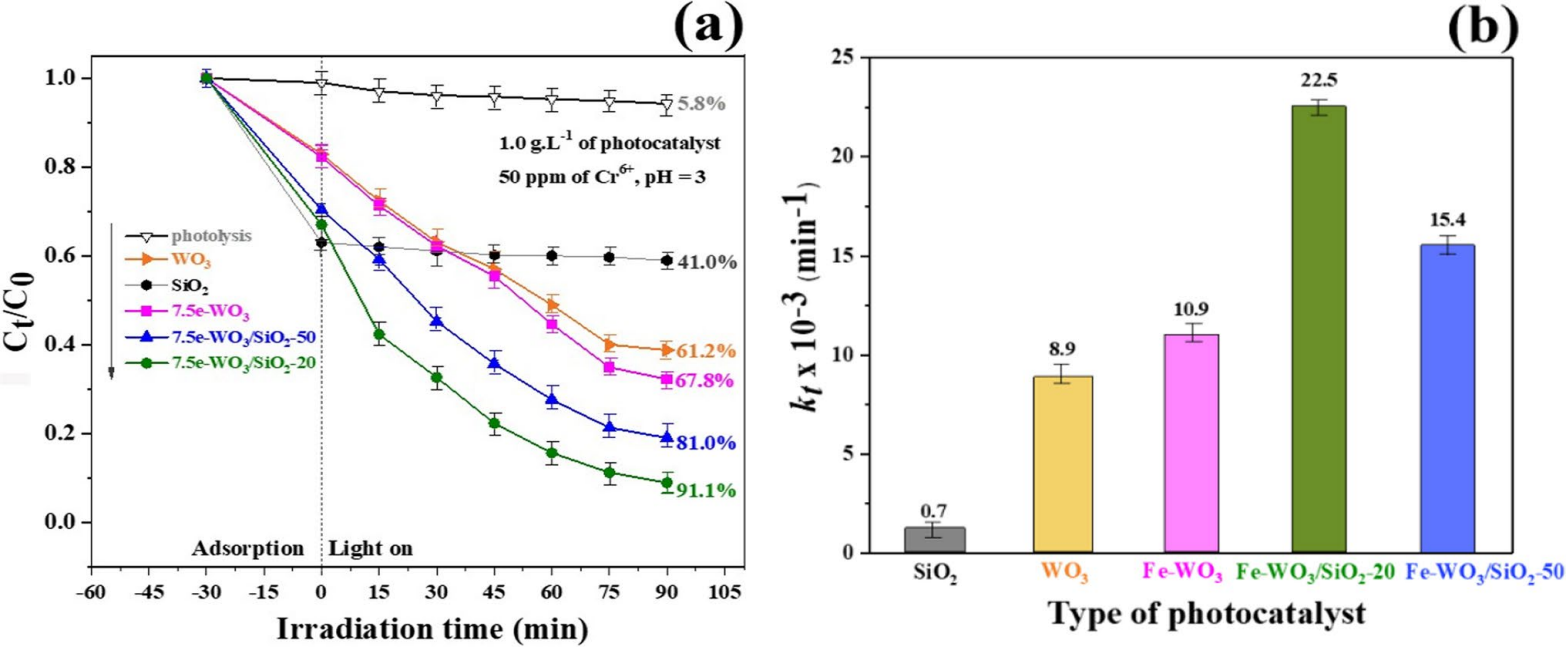
**a** Photoreduction of Cr^6+^ over overall catalyst and **b** first-order rate constant (*k*_*t*_) of over samples

The photocatalytic reduction of the concentrations was performed by varying the overall synthesised photocatalysts. The SiO_2_, WO_3_, 7.5% Fe-WO_3_, 7.5% Fe-WO_3_/SiO_2_-20, and 7.5% Fe-WO_3_/SiO_2_-50 heterostructures were obtained over 50 ppm of the Cr^6+^ concentration with a photocatalyst dosage of 1.0 g·L^−1^ and a pH of 3.0 under 90 min of visible light illumination at room conditions. Figure 8a represents the photoreduction of Cr^6+^ efficiencies over visible light illumination over overall catalysts, and the first-order rate constants (*k*_*t*_) were calculated and are similarly displayed in Fig. 8b. Moreover, the effect of photocatalyst dosage, pH of the suspension was also evaluated via the optimal result from %PE by using various type of photocatalyst. Furthermore*,* the usability test of photoreduction via the superior PE photocatalyst had studied together. The deactivation of used catalyst was confirmed by XRD patterns and FE-SEM after 5 cycles run.

The evaluation of the %PE and *k*_*t*_ of the Cr^6+^ reduction with the error bars of the synthesised photocatalysts is illustrated in Fig. 8a and b, respectively. Prior to %PE determination, 7.5% Fe-doped WO_3_/SiO_2_-20 demonstrated the highest Cr^6+^ removal activity of 91.1% within 90 min. The improved photocatalytic efficiency of the WO_3_/SiO_2_ nanocomposite was due to the presence of SiO_2_ NPs. As shown in Fig. 8a, it could be assumed from the evidence that by adding SiO_2_ to the composite, SiO_2_ acted as an adsorbent, which sufficiently enlarged the surface area of WO_3_, representing the main role of SiO_2_ as a high-surface-area catalyst support to generate well-dispersed Fe-WO_3_ NPs. The increased surface area of WO_3_ can facilitate the adsorption of organic pollutants through the formation of more useful adsorption**-**degradation sites [53]. Additionally, an Fe-WO_3_/SiO_2_ nanocomposite is a powerful photocatalyst for separating photogenerated electrons and holes, which is significant for photocatalytic activity. However, further adding SiO_2_ reduced the activity to 81.0%, presumably since the composite catalyst contained silicon as the main element. Silicon can significantly decrease the crystallinity of WO_3_ in composite materials by acting as a WO_3_ grain growth inhibitor [54]. The calculated results of the first-order rate constant (*k*_*t*_) versus irradiation time are shown in Fig. 8b and listed in Table 2. The largest rate constant of 22.5 × 10^–3^ min^−1^ was observed for the 7.5% Fe-WO_3_/SiO_2_-20 nanocomposite, which was larger than that of 7.5% Fe-WO_3_ without SiO_2_ (10.9 × 10^–3^ min^−1^). Thus, the 7.5 mol% Fe-doped WO_3_-20 catalyst was chosen for further investigation of the effects of various parameters on photoreduction efficiency. Furthermore, an evaluation of the photocatalytic reduction of the Cr^6+^ solution influenced by two factors, namely the photocatalyst dosage and the pH of the suspension, was carried out. Prior to %PE determination, the 7.5% Fe-doped WO_3_/SiO_2_-20 demonstrated the highest Cr^6+^ removal activity at 91.1% within 90 min.

**Table 2:** Comparison of photocatalytic reduction of Cr^6+^ solution over prepared nanomaterials

Photocatalysts	Cr^6+^ reduction activities (%)	Rate constant (*k*_*t*_) (× 10^−3^ min^−1^)	R^2^
Photolysis	5.8	0.3	0.98
SiO_2_	41.0	0.7	0.98
WO_3_	61.2	8.9	0.99
7.5% Fe-WO_3_	67.8	10.9	0.98
7.5% Fe-WO_3_/SiO_2_-50	81.0	15.4	0.99
7.5% Fe-WO_3_/SiO_2_-20	91.1	22.5	0.99

#### Effect of the photocatalyst dosage

**Fig. 9:**
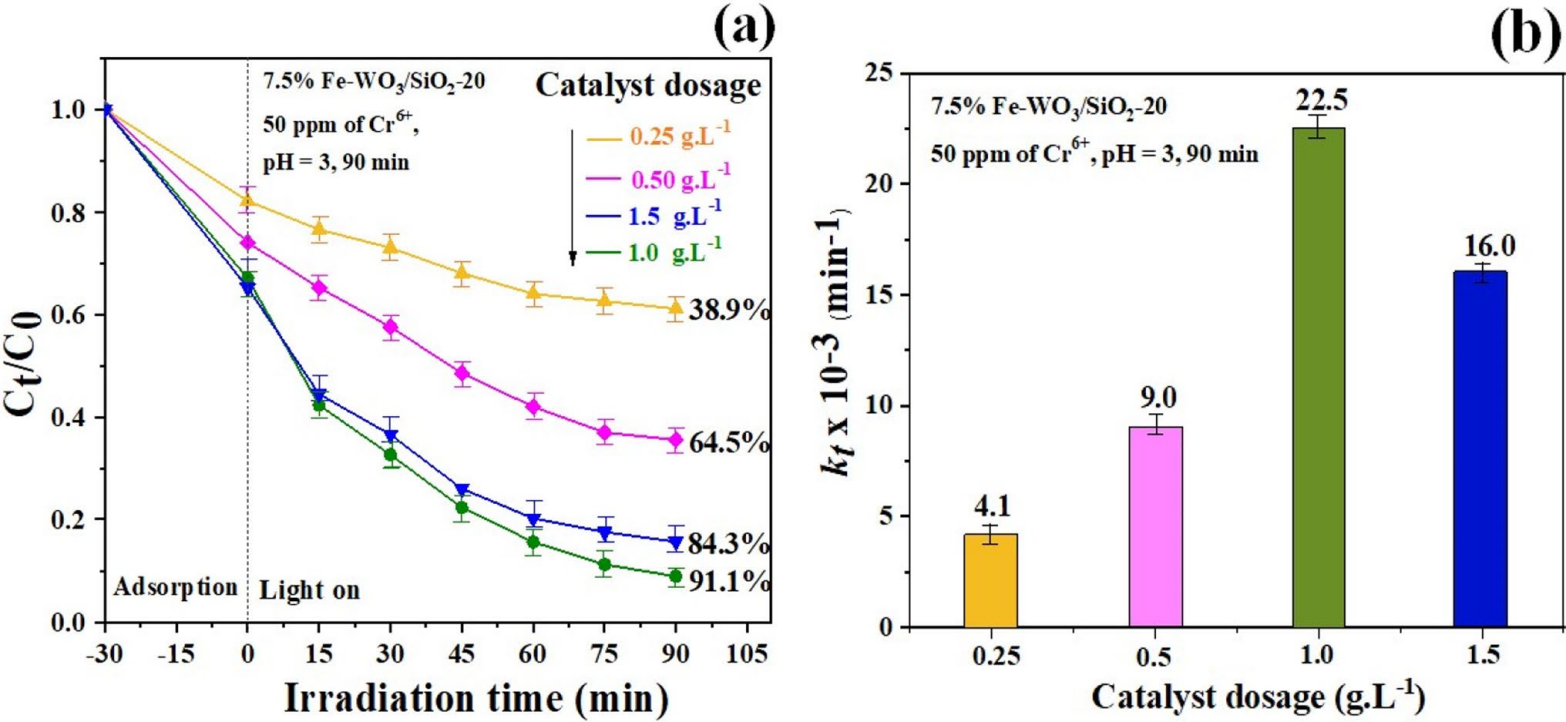
**a** Effect of the photocatalyst dosage on photoreduction of Cr^6+^efficiency over 7.5% Fe-WO3-SiO2-20 heterostructure photocatalyst **b** pseudo-first-order rate constant of (**a**)

The photocatalytic reduction of Cr^6+^ was conducted with the 7.5% Fe-WO_3_/SiO_2_-20 photocatalyst by varying the catalyst dosages at 0.25, 0.5, 1.0, and 1.5 g L^−1^, and the rate constant (*k*_*t*_) was also evaluated. As the % PE and *k*_t_ results in Fig. 9a–b illustrate, the %PE of the Cr^6+^ solution (50 ppm) decreased in the following order: 1.0 g L^−1^ (91.2%) > 1.5 g L^−1^ (86.8%), > 0.5 g L^−1^ (60.2%), and > 0.25 g L^−1^ (38.9%). The results revealed that with catalyst loading increments from 0.25 to 1.0 g, an increase in %PE was found, and after further increasing catalyst loading to 1.5 g·L^−1^, % PE was reduced to 86.6%. This was due to the increase in the catalyst content, which reached a higher quantity of active sites on the photocatalyst surface for the photoreduction reaction. However, upon increasing the catalyst loading to 2.0 g L^−1^, the photodegradation performance precipitously decreased due to the agglomeration and sedimentation of the catalyst particles, which caused an increase in the particle size and decrease in specific surface area, thus leading to a decrease in the number of active sites [55]. The excess catalyst content may have led to opaqueness and turbidity, possibly blocking the irradiation light, and increasing light scattering of the catalyst particles [56].

#### Effect of the pH of the suspension

**Fig. 10:**
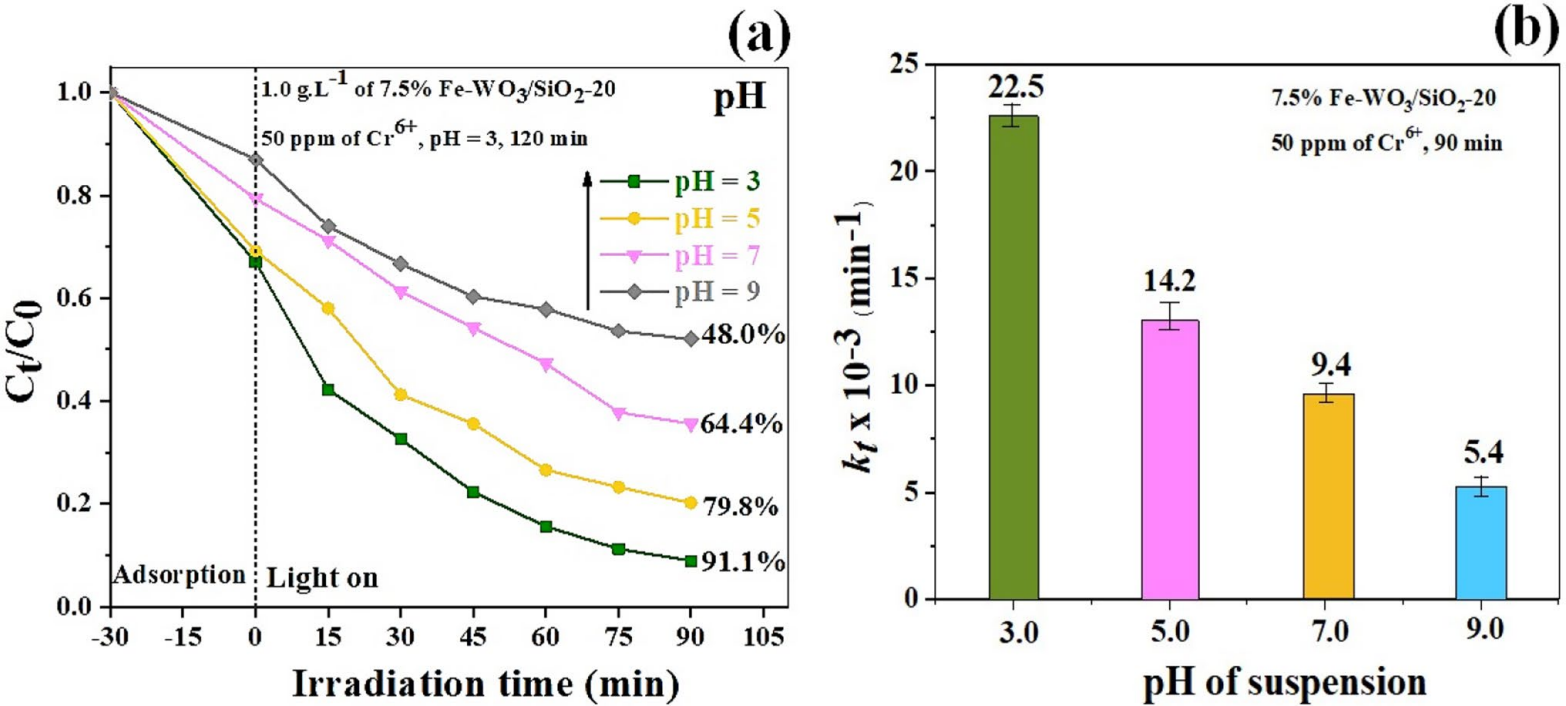
**a** Effect of the pH of suspension on photoreduction of Cr^6+^ efficiency over 7.5% Fe-WO_3_-SiO_2_-20 heterostructure photocatalyst **b** pseudo-first-order rate constant of (**a**)

The photocatalytic reduction performance of Cr^6+^ with the 7.5% Fe-WO_3_/SiO_2_-20 photocatalyst by changing the pH of the suspension to 3.0, 5.0, 7.0, and 9.0 was investigated, as shown in Fig. 10a–b. The optimum pH was 3.0, as it reached the highest reduction activity of 91.9% and 22.3 × 10^–3^ min^−1^ *kt* compared with suspensions with higher pH. The zeta potential value versus the pH of the catalyst suspension is displayed in Fig. S2. It can be seen that the PZC value of the 7.5% Fe-WO_3_/SiO_2_-20 photocatalyst suspension was approximately − 0.37 mV at pH 5.3. The catalyst surface revealed a negative charge below pH 5.3, and the electrostatic repulsion between the negative charge of the catalyst particles and the positive Cr^6+^ ions suggested the improvement of photocatalytic reduction activity [57]. Consequently, the surface of the nanocomposite was negatively charged at pH 3; therefore, the adsorption and enhanced photocatalytic reduction of the Cr^6+^ ions were strongly beneficial because of the strong electrostatic repulsion and charge attraction between the charges of the photocatalyst particles and the Cr^6+^ cations [58]. Therefore, the optimum condition of photocatalytic reduction of 50 ppm of the Cr^6+^ solution with the 7.5% Fe-WO_3_/SiO_2_-20 photocatalyst and 1.0 g L^−1^ catalyst loading at pH 3 illustrated the greatest efficiency for 90 min of visible light illumination.

**Fig. 11:**
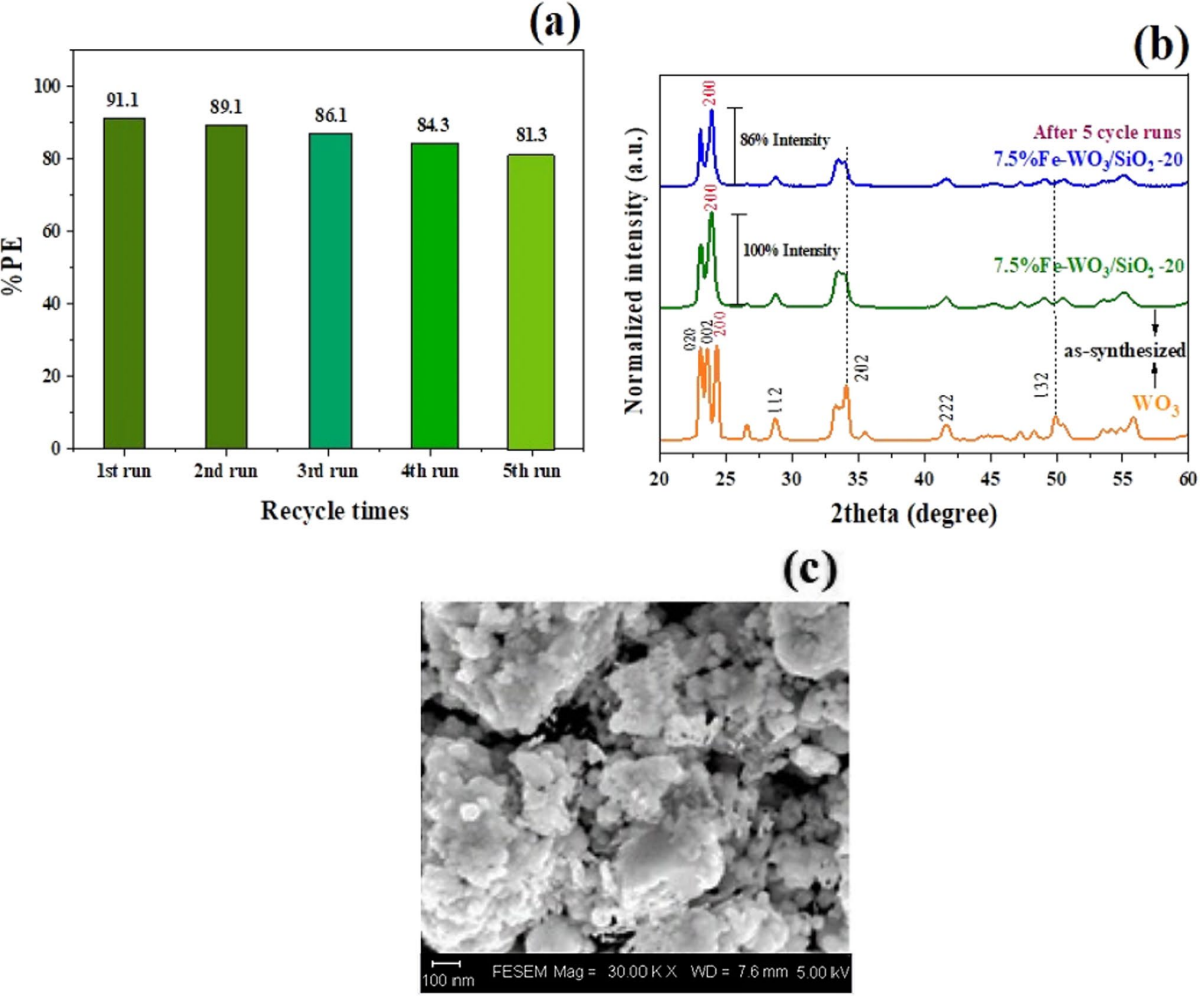
**a** Cyclic runs of photoreduction of 50 mg L^−1^ Cr^6+^ over 7.5% Fe-WO_3_-SiO_2_-20 heterostructure photocatalyst, **b** XRD patterns before and after recycling experiments for photoreduction of Cr(VI), and (c) FE-SEM image after the five recycles of 7.5% Fe-WO_3_-SiO_2_-20 heterostructure nanocomposite catalyst

The result from the cycling runs of the photoreduction of the Cr^6+^ solution with the 7.5% Fe-WO_3_/SiO_2_-20 nanocomposite under visible light irradiation and a catalyst loading of 1.0 g L^−1^ are demonstrated in Fig. 11a. The %PE demonstrated reduced only 9.3% after 5 cycle runs but fresh and but the spent-catalysed and WO_3_ products were also compared five times of 7.5% Fe-WO_3_/SiO_2_-20 sample a prepared fresh sample including the change XRD patterns crystallinity and 3D microstructure result from FE-SEM results. The XRD pattern of 5-spent time catalyst was demonstrated the good crystallinity, and XRD pattern of the used 7.5% Fe-WO_3_/SiO_2_-20 nanocomposite was found to decrease in the (202) peak, with an intensity of ~ 14% as shown in Fig. 11b. Meanwhile, Fig. 11c shows a minor change in noise pattern was observed the of the agglomeration of SiO_2_ NPs together with the rod-like WO_3_ like the fresh one in comparison with the un-catalysed nanocomposite.

However, the 5-spent times runs of the optimal catalyst observed a minor change of the photoreduction efficiency. There result indicates the 7.5% Fe-WO_3_/SiO_2_-20 does not suffer from the photocorrosive and chemical corrosive properties during the reduction towards Cr^6+^ reactions. Moreover, the leaching of Fe ions from the 7.5% Fe-WO_3_/SiO_2_-20 was not found in the supernatant of Cr^6+^ solution after determined by ICP-OES analysis [31]. These results demonstrate that the heterostructure photocatalyst exhibits stability against both photocorrosion and chemical corrosion in acidic conditions. Consequently, it offers superior stability and efficient reusability for removing Cr^6+^ effluent by using a retrievable Fe-doped WO_3_/SiO_2_-20% nanocomposite catalyst. This enhanced performance can be attributed to the inhibition of electron–hole recombination through the Fe^3+^ dopant and an increase in the active site surface after SiO_2_ loading [58]. Typically, the separation of e/h^+^ pairs occurs upon irradiation; however, e^−^/h^+^ is easily recombined without the charge carrier separation properties of a heterojunction. The PL spectra of SiO_2_, Fe-doped WO_3_, Fe-doped WO_3_/SiO_2_-50%, and 7.5% Fe-WO_3_/SiO_2_-20 are illustrated in Fig. S3. It is evident that 7.5% Fe-WO_3_/SiO_2_-20 emitted the lowest PL emission intensity in comparison to the other materials. This decrease in the PL spectra intensity indicates that the 7.5% Fe-WO_3_/SiO_2_-20 nanocomposite serves as the optimal catalyst, effectively suppressing electron–hole recombination in contrast to Fe-doped WO_3_, pure WO_3_, and pure SiO_2_.

**Fig. 12:**
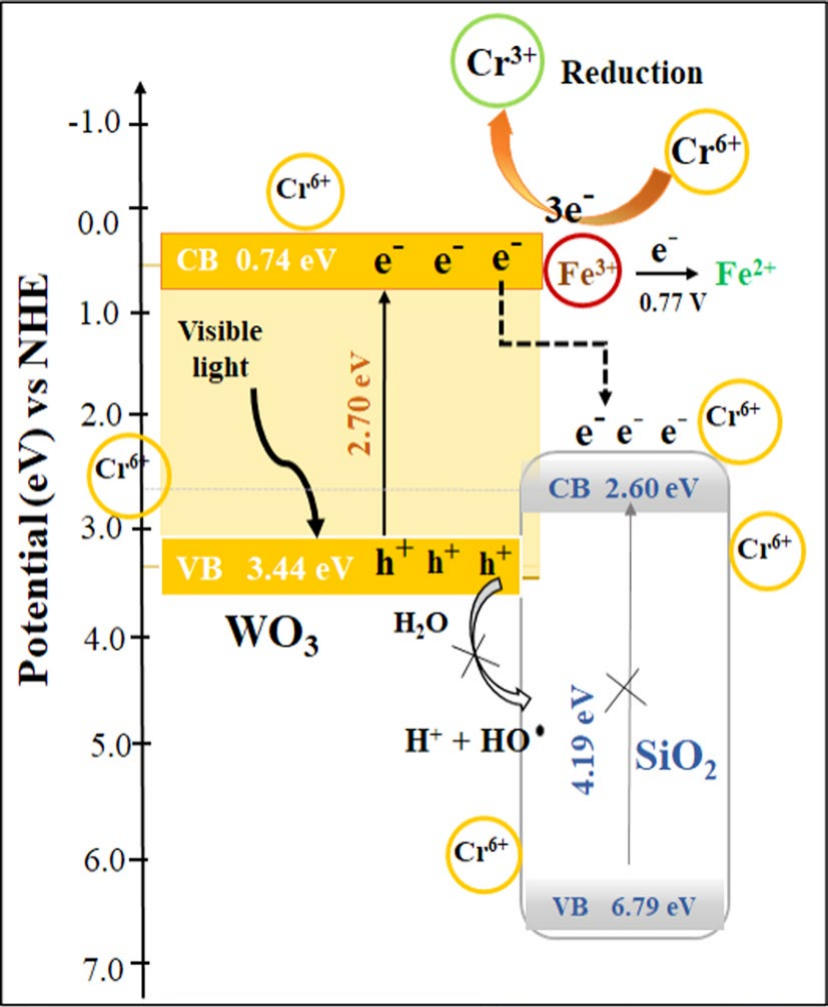
Possible mechanism (aliment at bulk ambient) of photocatalytic reduction of Cr^6+^ by the 7.5% Fe-WO_3_-SiO_2_-20 nanocomposite catalyst

Based on the results presented in Fig. 12, electron generation was observed to increase at the conduction band (CB) of the 7.5% Fe-doped WO_3_/SiO_2_-20% heterostructure. Inhibiting electron–hole recombination intensified the photoactivity of the WO_3_ catalyst. This, in turn, resulted in enhanced photoreduction and modified photocatalysis efficiency [59]. Table 3 compares the photocatalytic reduction of Cr^6+^ or organic dye activity of the prepared Fe-doped WO_3_ and WO_3_/SiO_2_ in various nanocomposites was compared with previous reports [13, 54, 60–63].

**Table 3:** Comparison of physiochemical properties and activities of photoreduction of Cr^6+^ solution and dyes, with different types and light sources of metal doped WO_3_/SiO_2_ photocatalysts

Catalyst type	Band gap (eV)	Light source	Reactant and concentration	Photocatalytic efficiency and reaction time	Pseudo-first-order rate (*k*_*t*_) (× 10^–3^ min ^−1^)	References
WO_3_/TiO_2_ Aerogel composite	2.60	130 W Xe	Cr (VI), 20 mg L^−1^	36.9%, 90 min	3.85	Yang et al. [13]
0.5% Au-SiO_2_-WO_3_ nanocomposite	2.80	72 W halogen	MB, 5.0 mg L^−1^	93%, and 51%, 200 min	8.70	De Puccio et al. [45]
5% Fe-WO_3_ nanoplate	2.50	300 W Xe	Cr (VI), 50 mg L^−1^	96.1%, 60 min	22.8	Feng et al. [60]
7.08% Fe-WO_3_	2.67	300 W Xe	Rh-B, 20 mg L^−1^	78.5%, 120 min	5.81	Song et al. [61]
3.7% Fe-SiO_2_-TiO_2_	N/A	Xe/Hg (UV region)	Cr (VI), 10 mg L^−1^	85%, 60 min	4.23	Elias [62]
5% Fe-doped WO_3_	2.39	500 W halogen	MB, 10 mg L^−1^	95%,120 min	22.7	Luxmi et al. [63]
(Fe^3+^/Fe^2+^)/SnO_2_/TiO_2_ composites	2.90	300 W Xe	Cr (VI), 20 mg L^−1^	90% 180 min	N/A	Venkatesh [64]
7.5% Fe-WO_3_/SiO_2_-20% nanocomposite	2.70	50 W Halogen	Cr (VI), 20 mg L^−1^	91.1%, 90 min	8.20	This study

The photocatalytic reduction performance of a photocatalyst depends on the intrinsic properties of NPs in the photocatalyst, including the band gap energy of each photocatalyst, light source, type of reactant, and the initial concentration of Cr^6+^ dye [65]. Song et al. [61] had explained that the generated electrons could be excited within the visible light spectrum, contributing to the practical enhancement of photocatalytic performance, as shown in Eqs. 5–8:

5 $${\text{WO}}_{{3}} + {\text{ h}}\upsilon \to {\text{e}}^{ - } + {\text{ h}}^{ + }$$

6 $${\text{Cr}}^{{{6} + }} + {\text{ 3e}}^{ - } \to {\text{Cr}}^{{{3} + }} :0.{\text{5 V NHE}}$$

7 $${\text{W}}^{{{6} + }} + {\text{e}}^{ - } \to {\text{W}}^{{{5} + }} :{\text{E}}^{0} {\text{cell = }} - 0.{\text{38 V NHE}}$$

8 $${\text{Fe}}^{{{3} + }} + {\text{e}}^{ - } \to {\text{Fe}}^{{{2} + }} :{\text{E}}^{0} {\text{cell}} = 0.{\text{77 V NHE}}$$

The potential mechanism for e^−^/h^+^ pairs separation at the 7.5% Fe-WO_3_-SiO_2_-20 surface under visible light irradiation is displayed in Fig. 12. As the results from calculated *E*_*g*_ result from Fig. 3b, the conduction band (CB) and valence band (VB) potentials of WO_3_ and SiO_2_ can be determined using Eq. 9 in our previous work:

9 $$E_{{{\text{CB}}}}^{0} = \chi {-} \, E^{C} {-} \, \frac{1}{2} \, E_{g}$$

*where χ* is the absolute electronegativity of the semiconductor (*χ* is 6.59 and 9.20 eV for WO_3_ and SiO_2_, respectively), *E*^*C*^ is the scaling factor relating the hydrogen electrode scale (NHE, bulk) to the absolute vacuum scale (AVS) (*ca.* 4.5 eV vs. NHE), and *E*_*g*_ is the band gap of Fe-WO_3_/SiO_2_-20 (2.70 eV) and SiO_2_ (4.19 eV) obtained in this study. Using the equations mentioned above, the CB position and VB edges of Fe-WO_3_/SiO_2_-20 were calculated to be 0.74 and 3.44 eV, respectively. For SiO_2_, the VB and CB positions potentials were determined to be 2.60 and 6.79 eV, respectively. However, photoreduction was only achievable at the CB of Fe-WO_3_/SiO_2_-20 due to visible light generated only a single WO_3_ semiconductor, which aligned the band gap energy. Moreover, the oxidation of h^+^ could not oxidised with the hydroxide ions and water molecules in the aqueous Cr^6+^ solution. Therefore, the potential of the accumulated electrons on CB of WO_3_ was located at a lower band position than the reduction potential of O_2_/O_2_^·^ (0.33 V vs. NHE) [66]. The results indicated an increase in the generation of electrons in the CB of the heterostructure and highlighted its crystallinity, adequate morphology, specific surface area, and extrinsic adsorption capacity [36]. The optimum loading of SiO_2_ in Fe-doped WO_3_ involved a concentration of 20 wt%. SiO_2_ supporter acted great supporter to WO_3_ NPs alike the type III heterojunction, and migration of charge carriers follows a similar pathway as in type II, resulting in no transference. However, SiO_2_ could help to trap Cr^6+^ ions on the interface and around the large surface area and high pore volume consisting of active surface under acidic condition. The photoinduced electrons initially reacted with the Cr^6+^ present near the periphery of the nanocomposite catalyst, as shown in Eq. 8. As shown in Eq. 6, these highly reactive protons and electrons cumulatively reduce Cr^6+^ to Cr^3+^. The purpose of the possible mechanism of the photocatalytic reduction of Cr^6+^ by the 7.5% Fe-WO_3_/SiO_2_-20 nanosized composite catalyst is presented in Fig. 12. The mechanism of (superoxide) radicals [66] involves the generation of Fe^3+^ ions; consequently, the reduction was controlled by electron capture. Furthermore, a smaller band gap implies a higher quantum efficiency for the material, enabling a greater number of electrons to cross the forbidden energy gap (band gap) into the CB. These electrons can be excited by photons in the visible light region, leading to an enhanced photocatalytic performance and abundant H_2_O molecules, producing hydroxyl radicals (·OH) and protons (H^+^).

The relative reduction potential of Fe^3+^/Fe^2+^ (Eq. 8) had a higher positive value than W^6+^/W^5+^ (Eq. 7). Thus, the Fe^3+^ doping in WO_3_ could prevent the self-corrosion of W^6+^. Then, the three generated e- reduced Cr^6+^ to Cr^3+^ as shown in Eq. 6. The standard electrode potential of Fe^3+^ (*E*^0^ cell = 0.77 V NHE) was higher than the CB potential, suggesting that a side photoreduction reaction also did not occur via the capture of e^−^ by Fe^3+^ ions (Eq. 8). Meanwhile, photocatalytic reduction at the CB of the WO_3_ surface particles occurred in the presence of visible light, and the transferred electrons from SiO_2_ generated electrons (e^−^) and supplied the valence band with holes (h^+^). In an aqueous system, h^+^ reacts with the photocatalytic reduction of toxic Cr^6+^ to less toxic Cr^3+^. Thus, Cr^6+^ ions diffused through the medium, representing the proposed mechanism of the photocatalytic reduction of Cr^6+^ with the 7.5% Fe-WO_3_/SiO_2_-20 nanocomposite catalyst [68].

## Conclusions

A visible light-driven 7.5% Fe-doped WO_3_/SiO_2_ nanocatalyst was successfully synthesised via a facile hydrothermal process coupled with the impregnation method. By applying 1.0 g L^−1^ of the 7.5% Fe-doped WO_3_/SiO_2_ composite catalyst for an illumination time of 90 min, a high pseudo-first-order kinetic rate led to an achievement of 91.1% photocatalytic activity. Photocatalytic activity depends on the intrinsic properties of a photocatalyst, such as a narrowed band gap and a small size. This study demonstrated a high degree of photocatalytic reduction of a hexavalent Cr^***6*+**^solution was achieved in this study. The reusable catalyst continued to exhibit high activity after a five-cycle run. Thus, we successfully synthesised a composite with enlarged activity, which can be attributed to three factors: (i) an extended visible light absorption region with narrowed band gap energy, (ii) a greater specific surface area (51.38 m^2^ g^−1^), and (iii) inhibition of the recombination of e^−^–h^+^ pairs, leading to electron charge separation due to the interactions between WO_3_ and SiO_2_ in the composite and electron trapping by doped Fe^3+^ ions. This was confirmed by the lowest absorption values obtained in the PL results. Therefore, the novel modified nanocomposite catalyst reported in this study may be promised for various environmental applications.

### Supplementary Information

Supplementary file.

## Data Availability

All generated and analysed data in this research included within the article can be used with no concern or Ethics. Additional Supplement Information 1 is attached file in the system.

## References

[1] Mohan D, Pittman CU (2006). Activated carbons and low cost adsorbents for remediation of tri- and hexavalent chromium from water. J Hazard Mater.

[2] Golonka MC (1995). Toxic and mutagenic effects of chromium (VI) a review. Polyhedron.

[3] Irshad MA, Irfan A, Zaki EM (2023). Enhancing chromium removal and recovery from industrial wastewater using sustainable and efficient nanomaterial: a review. Ecotoxicol Environ Saf.

[4] Gonzalez-Borrero PP, Sato F, Medina AN (2010). Optical band-gap determination of nanostructured WO3 film. Appl Phys Lett.

[5] Zheng J, Haider Z, Van T (2015). Tuning of the crystal engineering and photoelectrochemical properties of crystalline tungsten oxide for optoelectronic device applications. Cyst Eng Comm.

[6] Khanna A, Shetty V (2014). Solar light induced photocatalytic degradation of reactive Blue 220 (RB-220) dye with highly efficient Ag@TiO_2_ core–shell nanoparticles: a comparison with UV photocatalysis. Sol Energy.

[7] Nasuhoglu D, Yargeau V, Berk D (2011). Photo-removal of sulfamethoxazole (SMX) by photolytic and photocatalytic processes in a batch reactor under UV-C radiation (*λ*_max_ = 254 nm). J Hazard Mater.

[8] Wu Q, Xue S (2013). Photocatalytic reduction of Cr(VI) with TiO_2_ film under visible light. Appl Catal B.

[9] Ansari SA, Khan MM, Ansari O (2016). Nitrogen-doped titanium dioxide (N-doped TiO_2_) for visible light photocatalysis. New J Chem.

[10] Chang X, Sun S, Yin Y (2011). Solvothermal synthesis of Ce-doped tungsten oxide nanostructures as visible-light-driven photocatalysts. Nanotechnology.

[11] Tianjun N, Yang Z, Liu G (2020). N, Fe-doped carbon dot decorated gear-shaped W_O_3 for highly efficient UV-Vis-NIR-driven photocatalytic performance. Catalysts.

[12] Heri S, Alkian I, Mukholit M (2021). Analysis of Fe-doped ZnO thin films for degradation of rhodamine b, methylene blue, and *Escherichia coli* under visible light. Mater Res Express.

[13] Kumari H, Suman S, Ranga R (2023). A review on photocatalysis used for wastewater treatment: dye degradation. Water Air Soil Pollute.

[14] Lopez A, Marin M, Bosca F (2023). Synthesis and mechanistic insights of SiO_2_@WO_3_@Fe_3_O_4_ as a novel supported photocatalyst for wastewater remediation under visible light. Appl Mater Today.

[15] Balakumar V, Baishnisha A (2021). Rapid visible light photocatalytic reduction of Cr^6+^ in aqueous environment using ZnO-PPy nanocomposite synthesized through ultrasonic assisted method. Surf Interfaces.

[16] Zhang G, He H, Lu J (2018). Preparation of ZnIn_2_S_4_ nanosheet-coated CdS nanorod heterostructures for efficient photocatalytic reduction of Cr(VI). Appl Catal B.

[17] Islam B, Furukawa M, Kaneco S (2019). Formic acid motivated photocatalytic reduction of Cr(VI) to Cr(III) with ZnFe_2_O_4_ nanoparticles under UV irradiation. Environ Technol.

[18] Pudkon S, Kaowphong S, Hutchings G (2020). Enhanced visible-light-driven photocatalytic H_2_ production and Cr(VI) reduction of a ZnIn_2_S_4_/MoS_2_ heterojunction synthesized by the biomolecule-assisted microwave heating method. Catal Sci Technol..

[19] Pinto F, Wilson A, Kafizas A (2022). Systematic Exploration of WO_3_/TiO_2_ Heterojunction phase space for applications in photoelectrochemical water splitting. Phys Chem C.

[20] Yadav J, Singh JP (2022). WO_3_/Ag_2_S type-II hierarchical heterojunction for improved charge carrier separation and photoelectrochemical water splitting performance. J Alloys Compd.

[21] Yang L, Xiao Y, Liu SN (2010). Photocatalytic reduction of Cr(VI) on WO_3_ doped long TiO_2_ nanotube arrays in the presence of citric acid. Appl Catal B: Environ.

[22] Babyszko A, Wanag A, Sadłowski M (2022). Synthesis and characterization of SiO_2_/TiO_2_ as photocatalyst on methylene blue degradation. Catalysts.

[23] Phanichphant S, Nakaruk A, Chansaenpak K (2019). Evaluating the photocatalytic efficiency of the BiVO_4_/rGO photocatalyst. Sci Rep.

[24] Paola A, Lopez E, Marci G (2012). A survey of photocatalytic materials for environmental remediation. J Hazard Mater.

[25] Xie Y, Liu G, Cheng MJ (2012). Crystal facet-dependent photocatalytic oxidation and reduction reactivity of monoclinic WO_3_ for solar energy conversion. J Mater Chem.

[26] Ramkumar S, Rajarajan GJ (2016). Nitrogen-doped core–shell Fe/Fe_3_C@C nanocomposites for electromagnetic wave absorption. Mater Sci Mater Electron.

[27] Boonprakob N, Chen J, Inceesungvorn B (2014). Enhanced visible-light photocatalytic activity of g-C_3_N_4_/TiO_2_ films. J Colloid Interface Sci.

[28] Mugumo R, Ichipi E, Shepherd M, Tichapondwa NM (2023). Visible-light-induced photocatalytic degradation of rhodamine B dye using a CuS/ZnS p-n heterojunction nanocomposite under visible-light irradiation. Catalysts.

[29] Chang C, Zhu L, Fu Y (2013). Highly active Bi/BiOI composite synthesized by one-step reaction and its capacity to degrade bisphenol A under simulated solar light irradiation. Chem Eng J.

[30] Lu R, Zhong X, Tang M (2018). Effects of sintering temperature on sensing properties of WO_3_ and Ag-WO_3_ electrode for NO_2_ sensor. R Soc open sci.

[31] Chachvalvutikul A, Luangwanta T, Kaowphong S (2011). Double Z-scheme FeVO_4_/Bi_4_O_5_Br_2_/BiOBr ternary heterojunction photocatalyst for simultaneous photocatalytic removal of hexavalent chromium and rhodamine B. J Colloid Interface Sci.

[32] Acosta-Silva YJ, Nava R, Hernandez-Morales V (2011). Methylene blue photodegradation over titania-decorated SBA-15. Appl Catal B: Environ.

[33] Rasalingam S, Peng R, Koodali R (2014). Enhanced photocatalytic activity of W-doped and W-La-codoped TiO_2_ nanomaterials under simulated sunlight. J Nanomater.

[34] Kima K, Nam SK, Park JH (2019). Growth of BiVO_4_ nanoparticles on a WO_3_ porous scaffold: improved water-splitting by high band-edge light harvesting. J Mater Chem A.

[35] Shpak AP, Korduban AM, Medvedskij MM (2007). XPS studies of active elements surface of gas sensors based on WO_3−__*x*_ nanoparticles. J Electron Spectrosc.

[36] Eichhorn SJ, Sampson WW (2010). Relationships between specific surface area and pore size in electrospun polymer fibre networks. J R Soc Interface.

[37] Wetchakun N, Wanwaen P, Phanichphant S (2020). Correction: influence of Cu doping on the visible-light-induced photocatalytic activity of InVO_4_. RSC Advs.

[38] Joseph CG, Taufic YH, Musta B (2021). Application of plasmonic metal nanoparticles in TiO_2_–SiO_2_ composite as an efficient solar-activated photocatalyst: a review paper. Front Chem.

[39] Cui L, Wang Y, Kang S (2017). Facile preparation of Z-scheme WO_3_/g-C_3_N_4_ composite photocatalyst with enhanced photocatalytic performance under visible light. Appl Surface Sci.

[40] Hirata S, Inoue A, Inada M (2021). Synthesis and adsorption-photodecomposition properties of mesoporous SiO_2_-TiO_2_/WO_3_ composite. J Ceram Soc Japan.

[41] Chen S, Xiao Y, Xie W (2018). Facile strategy for synthesizing non-stoichiometric monoclinic structured tungsten trioxide (WO_3−__*x*_) with plasma resonance absorption and enhanced photocatalytic activity. Nanomaterials.

[42] Gui MS, Zhang WD, Su QX (2011). Preparation and visible light photocatalytic activity of Bi_2_O_3_/Bi_2_WO_6_ heterojunction photocatalysts. J Solid State Chem.

[43] Tao Y, Luca OD, Singh B (2020). WO_3_–SiO_2_ nanomaterials synthesized using a novel template-free method in supercritical CO_2_ as heterogeneous catalysts for epoxidation with H_2_O_2_. Mater Today Chem.

[44] Ghosh S, Acharya S, Tripathi D (2014). Preparation of silver–tungsten nanostructure materials for selective oxidation of toluene to benzaldehyde with hydrogen peroxide. J Mater Chem A.

[45] DePuccio D, Botella P, O’Rourke B (2015). Degradation of methylene blue using porous WO_3_, SiO_2_−WO_3_, and their Au-loaded analogs: adsorption and photocatalytic studies. ACS Appl Mater Interfaces.

[46] Can F, Courtois X, Duprez D (2021). Tungsten-based catalysts for environmental applications. Catalysts.

[47] Fernandes E, Gomes J, Martins RC (2022). Semiconductors application forms and doping benefits to wastewater treatment: a comparison of TiO_2_, WO_3_, and g-C_3_N_4_. Catalysts.

[48] Huang J, Tan G, Ren H (2014). Photoelectric activity of a Bi_2_O_3_/Bi_2_WO_6–__*x*_F_2__*x*_ heterojunction prepared by a simple one-step microwave hydrothermal method. Appl Mater Interfaces.

[49] Sokolov A, Filatova E, Afanasev V (2009). Interface analysis of HfO_2_ films on (1 0 0)Si using X-ray photoelectron spectroscopy. J Phys D Appl Phys.

[50] Wetchakun K, Wetchakun N, Sakulsermsuk S (2020). An overview of solar/visible light-driven heterogeneous photocatalysis for water purification: TiO_2_- and ZnO-based photocatalysts used in suspension photoreactors. Int J Ind Chem.

[51] Channei D, Inceesungvorn B, Wetchakun N (2017). Photocatalytic degradation of methyl orange by CeO_2_ and Fe-doped CeO_2_ films under visible light irradiation. Sci Rep.

[52] El-Nemr MA, Aigbe UO, Ukhurebor KE (2022). Adsorption of Cr^6+^ ion using activated *Pisum sativum* peels-triethylenetetramine. Environ Sci Pollut Res.

[53] Brady RL, Southmayd D, Contescu C (1991). Surface area determination of supported oxides: WO_3_/Al_2_O_3_. J Catal.

[54] Wang J, Ren J, Yao H (2016). Synergistic photocatalysis of Cr(VI) reduction and 4-chlorophenol degradation over hydroxylated α-Fe_2_O_3_ under visible light irradiation. J Hazard Mater.

[55] Channei D, Chansaenpak K, Phanichphant S (2021). Synthesis and characterization of WO_3_/CeO_2_ heterostructured nanoparticles for photodegradation of indigo carmine dye. ACS Omega.

[56] Li Y, He H, Li J (2015). Fast photocatalytic degradation of dyes using low power laser-fabricated Cu_2_O–Cu nanocomposites. RSC Adv.

[57] Yu J, Xiang Q, Zhou M (2009). Preparation characterization and visible-light-driven photocatalytic activity of Fe-doped titania nanorods and first-principles study for electronic structures. Appl Catal B Environ.

[58] Samanta S, Srivastava R (2017). Thermal catalysis vs. photocatalysis: A case study with FeVO_4_/g-C_3_N_4_ nanocomposites for the efficient activation of aromatic and benzylic C–H bonds to oxygenated products. Appl Catal B Environ.

[59] Shi F, Bai L, Liu J (2012). Preparation and characterization of SiO_2_–WO_3_ composite aerogel by ambient pressure drying process. Adv Mat Res.

[60] Feng M, Liu Y, Zhao Z (2019). The preparation of Fe doped triclinic-hexagonal phase heterojunction WO_3_ film and its enhanced photocatalytic reduction of Cr (VI). Mater Res Bull.

[61] Song H, Li Y, Lou Z (2015). Synthesis of Fe-doped WO_3_ nanostructures with high visible-light-driven photocatalytic activities. Appl Catal B Environ.

[62] Elias N, Ullah S, Perissinotto AP (2021). Thermally stable SiO_2_@TiO_2_ core@shell nanoparticles for application in photocatalytic self-cleaning ceramic tiles. Mater Adv.

[63] Luxmi V, Kumar A (2019). Enhanced photocatalytic performance of *m*-WO_3_ and *m*-Fe-doped WO_3_ cuboids synthesized via sol-gel approach using egg albumen as a solvent. Mater Sci Semicond Process.

[64] Venkatesh D, Anbalagan K (2023). Interfacial charge transfer of hybrid (Fe^3+^/Fe^2+^)/SnO_2_/TiO_2_ composites for highly efficient degradation of chromium (VI) under ultra-violet light illumination via Z-scheme. Opt Mater.

[65] Yu J, Xiang Q, Zhou M (2009). Preparation, characterization and visible-light-driven photocatalytic activity of Fe-doped titania nanorods and first-principles study for electronic structures. Appl Catal B Environ.

[66] Wood PM (1987). The two redox potentials for oxygen reduction to superoxide. Trends Biochem Sci.

