# Supplementary material for: High-performance photocatalytic reduction of Cr(VI) using a retrievable Fe-doped WO3/SiO2 heterostructure
Source: Discov Nano. 2024 Jan 31;19(1):22. doi: 10.1186/s11671-023-03919-0 (PMC10831000; doi:10.1186/s11671-023-03919-0)
Supplement: Supplementary file 1 — Supplementary file. [file 11671_2023_3919_MOESM1_ESM.docx]

**Supplementary Information (SI)**

**High performance photocatalytic reduction of Cr(VI) using
a retrievable Fe-doped WO_3_/SiO_2_ heterostructure**

Natkritta Boonprakob^1*^, Duangdao Channei^2^ and Chen Zhao^3^

^1^ Program of Chemistry, Faculty of Science and Technology, Uttaradit Rajabhat University, Uttaradit, 53000, Thailand.

^2^ Department of Chemistry, Faculty of Science, Naresuan University, Phitsanulok, 65000, Thailand.

^3^ School of Materials and Energy, Guangdong University of Technology, Guangzhou, 510006, PR. China.

Corresponding author Phone: +66 8892 6149

E. Mail: natkritta.boo@uru.ac.th

**Figures**


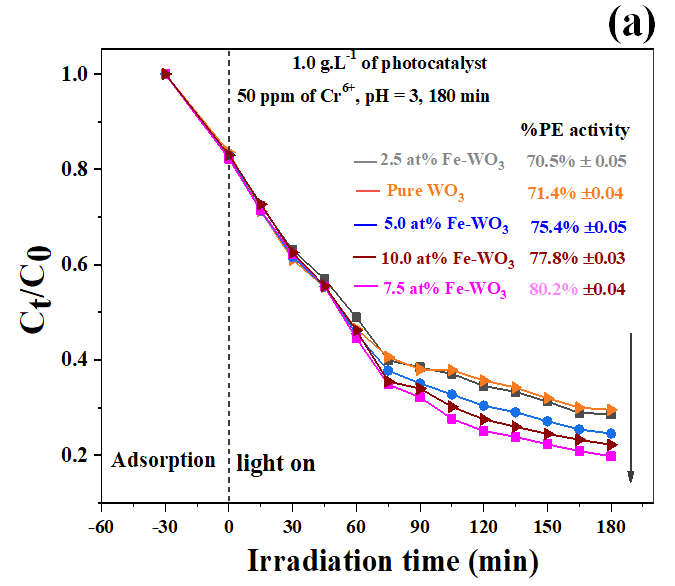

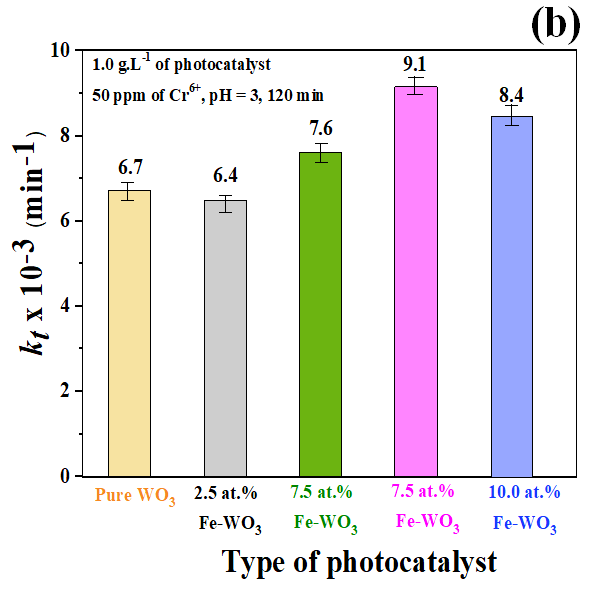


**Fig. S1** (a) Photocatalytic reduction activities and (b) kinetic rate constant (*k_t_*) of Fe-WO_3_ varying in the range of 2.5–10.0 mol% Fe^3+^.

Fig. S1(a) shows the visible light-driven photocatalytic reduction of Cr^6+^ over Fe-doped WO_3_ at various contents of Fe^3+^ under the condition of 1.0 g·L^-1^ of catalyst dosage, 20 ppm Cr^6+^ solution, 50-watt halogen light source, and 3.0 pH. The 7.5 mol% Fe-doped WO_3_ catalyst exhibited the highest efficiency of Cr^6+^ removal, *ca.* 80.2% within 180 min, whereas the photoreduction performances of SiO_2_ and WO_3_ were 41.0 and 71.4%, respectively. The kinetic constant rate (*k_t_*) of Fe-WO_3_ in the range of
2.5–10.0 mol% are displayed in Fig. S1(b). The highest rate constant of 9.1 🗙 10^-3^ min^−1^ was observed for the 7.5% Fe-WO_3_ nanocomposite. Therefore, the 7.5 mol% Fe-doped WO_3_ catalyst was selected for further investigation. SiO_2_ was then added at 20 and 50 wt% to yield 7.5% Fe-doped WO_3_/SiO_2_-20 and WO_3_/SiO_2_-50 nanocomposites.


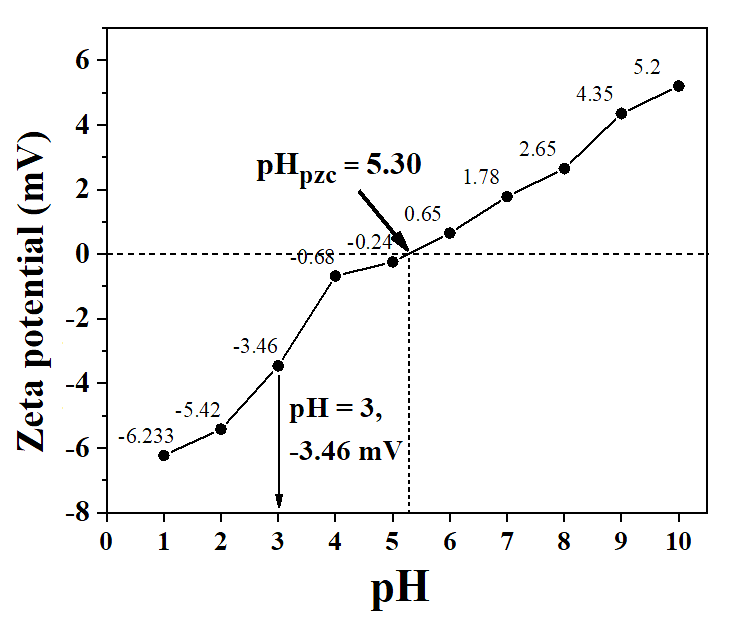


**Fig. S2** Zeta potential values of optimum photocatalyst vs pH of catalyst suspension.


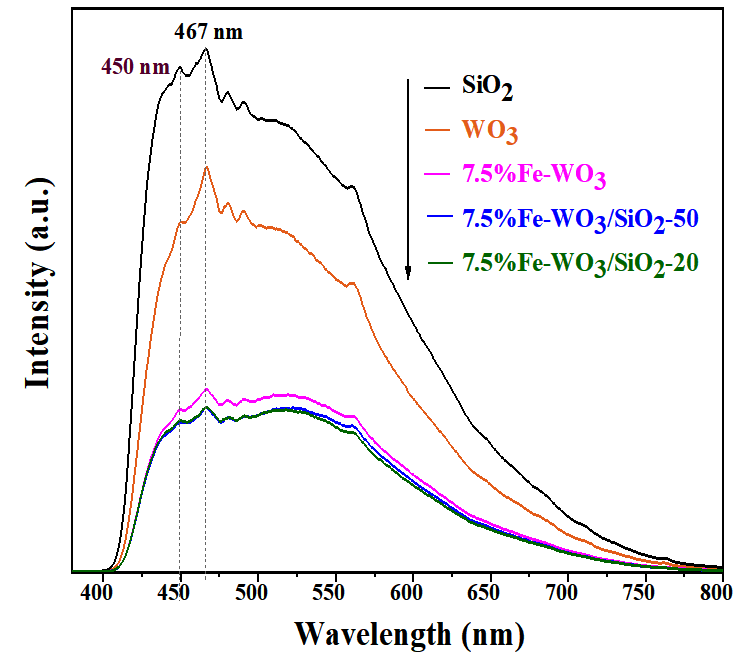


**Fig. S3** PL spectra of obtained photocatalysts.
